# Comprehensive metabolic profiling of the new designer stimulant MDPiHP—in vitro and in vivo identification of potential biomarkers for detection in human samples

**DOI:** 10.1007/s00216-026-06417-1

**Published:** 2026-03-12

**Authors:** Aurora Balloni, Johannes Kutzler, Giuseppe Basile, Francesco P. Busardò, Jeremy Carlier, Volker Auwärter

**Affiliations:** 1 Institute of Forensic Medicine, Forensic Toxicology, Medical Center – University of Freiburg, Faculty of Medicine, University of Freiburg, Freiburg, Germany; 2 Department of Biomedical Sciences and Public Health, Section of Legal Medicine, Unit of Forensic Toxicology, Marche Polytechnic University, Ancona, Italy; 3 Hermann Staudinger Graduate School, University of Freiburg, Freiburg, Germany

**Keywords:** MDPiHP, 3,4-Methylenedioxy-α-pyrrolidinoisohexanophenone, Synthetic cathinone metabolism, Metabolite identification, Biomarkers of intake

## Abstract

New psychoactive substances (NPS) pose an increasing threat to public health. Synthetic cathinones are among the most prevalent subclasses of NPS in Europe. Recently, MDPiHP (3,4-methylenedioxy-α-pyrrolidinoisohexanophenone), a positional isomer of MDPHP (3,4-methylenedioxy-α-pyrrolidinohexanophenone), has been detected in several seizures across Europe. However, its pharmacological and toxicological profiles remain unknown. In this study, we report the first investigation of MDPiHP metabolism, combining in vitro pooled human liver microsome (pHLM) experiments and the analysis of authentic human biological samples, including urine, blood, serum, and plasma. High-performance liquid chromatography coupled with quadrupole time-of-flight mass spectrometry (HPLC-QToF-MS) enabled the tentative identification of eleven metabolites in the in vitro assay. The most abundant metabolite was dimethyl-MDPiHP (M8), followed by dihydro-MDPiHP (M1.2) and hydroxy-MDPiHP (M2.3). In human biological samples, 22 metabolites were detected. The most abundant were dihydro-MDPiHP (M1.2) and M3, generated through pyrrolidine ring hydroxylation followed by ring opening and terminal carboxylation. Together with the parent compound, these metabolites are proposed as biomarkers of intake. A limitation of this study is the known instability of synthetic cathinones in biological matrices. This may alter metabolite ratios, and consequently, parent compound levels may be underestimated. A comparison between in vitro and in vivo findings revealed differences in the formation of metabolites, underscoring the limitations of the pHLM model in fully reproducing human metabolic complexity. The comprehensive metabolic profile described provides essential information for clinical and forensic toxicology, enabling the reliable detection of MDPiHP use and laying the groundwork for further studies on its toxicity and pharmacokinetics.

**Graphical Abstract:**

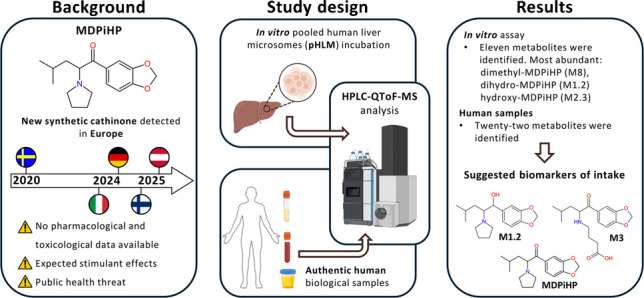

**Supplementary Information:**

The online version contains supplementary material available at 10.1007/s00216-026-06417-1.

## Introduction

New psychoactive substances (NPS) are a broad class of compounds intentionally designed to mimic the effects of controlled drugs. Over the past decade, the NPS market has evolved rapidly, posing substantial challenges to public health and law enforcement agencies worldwide [1, 2]. Among NPS, synthetic cathinones (SCat) represent a prominent subclass, particularly prevalent across Europe and Central Asia. Analysis of seizure data from 2019 to 2023 reveals that SCat accounted for approximately 21% of synthetic NPS seizures, ranking second only to synthetic cannabinoids. Notably, most SCat seizures took place in Europe (79%), with Eastern Europe accounting for 68% of these cases. While recent reports indicate a decline in SCat seizures in some regions, likely reflecting effects of enhanced legal controls under amphetamine-type stimulant legislation, evidence indicates that global markets continue to expand, as reflected in rising seizure rates within this drug class [2].

Within the subclass of SCat, pyrrolidinophenones have emerged as a substantial public health concern due to their widespread availability and potent psychostimulant effects [1]. These compounds primarily act as inhibitors of dopamine and norepinephrine reuptake transporters, while exerting a comparatively low influence on serotonin reuptake [3, 4]. Common adverse effects associated with SCat use include tachycardia, hypertension, hyperthermia, and agitation, reflecting their stimulant pharmacodynamics [5–7].

MDPiHP (IUPAC name: 1-(2*H*−1,3-benzodioxol-5-yl)−4-methyl-2-(pyrrolidin-1-yl)pentan-1-one) is a pyrrolidinophenone-type cathinone and a positional isomer of MDPHP (IUPAC name: 1-(2*H*−1,3-benzodioxol-5-yl)−2-(pyrrolidin-1-yl)hexan-1-one) [8]. Structurally, MDPiHP shares considerable similarity with internationally controlled cathinones such as MDPV and α-PVP. Despite its increasing identification in seizures across Europe between 2020 and 2025, covering Sweden, Slovenia, Italy, Germany, Finland, and Austria, and its legal control in countries like Italy, Lithuania, and Norway, limited information is available regarding its pharmacology and toxicology. Given its structural similarities to potent stimulants, MDPiHP is presumed to exert stimulant effects. Several cases describing acute intoxications characterized by agitation, tachycardia, and hypertension have been reported [6, 7, 9]. However, no experimental data have yet been published. Metabolic studies on MDPHP revealed phase I metabolites formed via hydroxylation and demethylation pathways, suggesting hepatic biotransformation [9–11].

It is important to note that positional isomers may display distinct pharmacokinetic and pharmacodynamic profiles. Variations in side chain branching could not only influence receptor binding/affinity and interaction with transporters, but also affinity to metabolizing enzymes. This potentially affects metabolite profiles, thus warranting a systematic investigation of metabolism. Considering the rising prevalence and the potential health risks of this compound, detailed metabolic and toxicological studies are essential to assist forensic toxicologists in its detection and monitoring. Such information would also support abstinence control and clinical toxicology.

To address this knowledge gap, the present study aimed to elucidate the metabolism of MDPiHP for the first time. This was achieved through in vitro pooled human liver microsome (pHLM) assays alongside analysis of human biological specimens, including urine, blood, serum, and plasma. The identification of specific metabolic markers provides useful tools for the detection of MDPiHP consumption.

## Materials and methods

The in vivo and in vitro metabolism of MDPiHP was investigated. Human biological samples were submitted in-house to the Institute of Forensic Medicine, Forensic Toxicology, Medical Center – University of Freiburg for routine toxicological analysis of designer stimulants. No personal or identifying data such as gender, age, body weight, amount and timing of intake, co-ingestion of other substances, or storage conditions was available. The analyses were solely performed at the client’s request for a routine toxicological analysis. Therefore, no ethical approval was required. Human samples as well as pHLM incubations were analyzed using liquid chromatography quadrupole time-of-flight mass spectrometry (LC-QToF-MS). To complement the experimental findings, an in silico prediction of the metabolic pathway of MDPiHP was also performed.

### Chemicals and reagents

All solvents were of liquid chromatography–mass spectrometry (LC–MS) grade. Methanol and acetonitrile were sourced from Sigma-Aldrich (Steinheim, Germany), while isopropanol and dichloromethane were obtained from VWR and Carl Roth (Karlsruhe, Germany), respectively. Deionized water was freshly produced in-house using a Medica® Pro deionizer from ELGA (Celle, Germany). Formic acid (98%) and hydrochloric acid (37%) were purchased from Carl Roth (Karlsruhe, Germany), while acetic acid (98%) and ammonia solution (25%) were sourced from Merck (Darmstadt, Germany). Phosphate buffer solutions at pH 6.0 and pH 7.4 were prepared prior to use, and ammonium formate (10 M, 99.995%) was acquired from Sigma-Aldrich (Steinheim, Germany). The analytical reference standards of MDPiHP and the internal standard PCP-d_5_ were obtained from Cayman Chemical (Ann Arbor, Michigan, USA). Pooled human liver microsomes (pHLMs, 150 donors, 20 mg protein/mL in 250 mM sucrose) were obtained from Corning (New York, USA). NADPH-regenerating solutions were also procured from Corning, consisting of solution A (26 mM NADP^+^, 66 mM glucose-6-phosphate, and 66 mM magnesium chloride in water) and solution B (40 U/mL glucose-6-phosphate dehydrogenase in 5 mM sodium citrate, with a reductase activity of 0.43 μmol/min × mL). For liquid chromatography tandem mass spectrometry (LC–MS/MS) analysis, the mobile phases consisted of 2 mM ammonium formate in deionized water with 1% acetonitrile and 0.1% formic acid (mobile phase A) and 2 mM ammonium formate in acetonitrile with 0.1% formic acid (mobile phase B). For liquid chromatography–quadrupole time-of-flight mass spectrometry (LC-QToF-MS) analysis, the mobile phases consisted of 2 mM ammonium formate in deionized water with 1% methanol and 0.1% formic acid (mobile phase C) and 2 mM ammonium formate in methanol with 0.1% formic acid (mobile phase D). The sodium formate/acetate clusters solution used for external and internal mass calibration of the QToF/MS instrument was prepared by mixing 250 mL deionized water, 250 mL isopropanol, 750 µL acetic acid, 250 µL formic acid, and 500 µL of 1 M sodium hydroxide.

### Human samples preparation

Blood, serum, plasma, and urine samples were prepared using solid-phase extraction according to a previously described method [12]. Briefly, 10 µL of PCP-d_5_ solution (0.25 µg/mL) was added as an internal standard to 0.5 mL of each sample, followed by the addition of 2.5 mL of phosphate buffer (pH 6). The resulting mixture was mixed thoroughly to ensure homogeneity. Solid-phase extraction was carried out using an automated system (Aspec® GX-274, Gilson, Middleton, Wisconsin, USA) equipped with Chromabond® Drug cartridges (Macherey–Nagel, Düren, Germany). Prior to sample loading, the cartridges were conditioned with 2 mL of methanol and subsequently equilibrated with 2 mL of phosphate buffer (pH 6). After conditioning, the samples were loaded onto the cartridges, and three sequential washing steps were performed using 1 mL of water, 1 mL of 0.1 M acetic acid, and 2 mL of methanol. Analytes were eluted with 1.5 mL of a dichloromethane/isopropanol/25% ammonia solution (80/20/2, v/v/v). The eluates were evaporated to near dryness under a gentle nitrogen stream at 40 °C. To prevent the loss of volatile amines during this evaporation step, 100 µL of a hydrochloric acid (37%)/isopropanol solution (1/3, v/v) was added prior to evaporation to complete dryness. Finally, the residues were reconstituted in 100 µL of mobile phase consisting of 99% mobile phase A (2 mM ammonium formate in deionized water with 1% acetonitrile and 0.1% formic acid) and 1% mobile phase B (2 mM ammonium formate in acetonitrile and 0.1% formic acid).

### Quantification of MDPiHP by LC–MS/MS

The LC–MS/MS system used for analysis consisted of a Shimadzu Prominence HPLC setup, comprising three LC-20AD SP pumps, a DGU-20A3 degasser, a SIL-20AC autosampler (set to 10 °C with an injection volume of 10 µL), a CTO-20AC column oven (maintained at 30 °C), and a CBM-20A controller. Chromatographic separation was achieved using a Kinetex® Biphenyl column (100 × 2.1 mm, 2.6 μm particle size, Phenomenex, Aschaffenburg, Germany) equipped with a corresponding guard column (SecurityGuard™ ULTRA Cartridges UHPLC Biphenyl for 2.1 mm internal diameter columns). A gradient elution schedule was applied using mobile phases A and B. The starting condition consisted of 5% B, which was held for 3 min. From 3 to 8 min, the proportion of B was increased to 15% and maintained until 11 min. Between 11 and 18 min, the proportion of B was further raised to 80%, followed by a rapid increase to 95% at 18.5 min. This highly organic composition was held constant until 21 min. Finally, the system was returned to the initial condition of 5% B at 21.5 min, which was held for an additional 2.5 min to ensure equilibration. The LC flow rate was set to 0.3 mL/min, and an additional post-column infusion of isopropanol with a flow rate of 0.1 mL/min was introduced to enhance signal intensity.

The HPLC system was coupled to a Sciex Triple Quad 6500+ mass spectrometer equipped with a TurboIonSpray® interface (Sciex, Darmstadt, Germany). The mass spectrometer operated in positive electrospray ionization (ESI) mode, and data acquisition was performed in multiple reaction monitoring (MRM) mode using Analyst® software (version 1.5.1). Optimized MS parameters for the detection of MDPiHP are summarized in Table 1. The ion source conditions were as follows: collision gas pressure of 6 psi (41 kPa), curtain gas pressure of 30 psi (270 kPa), ion spray voltage of 4000 V, and source temperature of 550 °C.

**Table 1:** Ion transitions of MDPiHP and internal standard PCP-d_5_ along with MS parameters optimized for the target compound

	Precursor ion *m/z*	Product ion *m/z*	RT (min)	DP (V)	EP (V)	CE (V)	CXP (V)
MDPiHP	290.2	135	14.59	65	10	37	6
		219.1	14.59	65	10	27.5	4
PCP-d_5_	249.1	164.1	15.10	50	6	20	14

*CE*, collision energy; *CXP*, cell exit potential; *DP*, declustering potential; *EP*, entrance potential; *RT*, retention time

For quantification, calibration samples were prepared by spiking 0.5 mL of blank urine with 10 µL of internal standard solution and appropriate volumes of MDPiHP reference solution at concentrations of either 0.5 µg/mL or 5 µg/mL. This resulted in six calibration levels ranging from 1 to 50 ng/mL (1, 2, 5, 10, 20, and 50 ng/mL). Additionally, a negative control sample was prepared using 0.5 mL of blank urine spiked with 10 µL of the internal standard.

Method validation for MDPHP was previously performed in accordance with the guidelines of the German Society of Toxicological and Forensic Chemistry (GTFCh), confirming that the method was both selective and specific [9, 13, 14]. A short cross-validation of MDPiHP and MDPHP showed identical responses and equal selectivity and specificity. Therefore, concentrations of MDPiHP are given as semi-quantitative values (approx.). However, as MDPHP—an isomer of MDPiHP—could potentially interfere when present simultaneously, sufficient chromatographic resolution of these two compounds is critical. The peak resolution between MDPiHP and MDPHP was determined to be 1.14, corresponding to a retention time difference of 0.12 min (Figure S1, Online Resource 1). Although this resolution was sufficient to allow for peak identification, the presence of both analytes in the same sample may still affect quantitative results due to chromatographic peak overlap.

### Pooled human liver microsome (pHLM) assay procedure

Incubation with pHLMs was performed as previously described [15] with minor modifications. Briefly, the incubation mixture consisted of 68 µL deionized water, 20 μL 0.5 M phosphate buffer (pH 7.4), 5 μL NADPH-regenerating solution A, 1 μL NADPH-regenerating solution B, and 1 μL MDPiHP (1 mg/mL stock solution in methanol). This mixture was combined with 5 μL of pHLM solution. The incubations were conducted at 37 °C and terminated at defined time points (30 min, 1 h, and 2 h) by the addition of 300 μL of ice-cold acetonitrile. To improve phase separation prior to centrifugation, 50 μL of 10 M ammonium formate was added. Subsequently, the samples were centrifuged (4000 rpm, 10 min) and the resulting organic phase was collected and stored at −20 °C until analysis. Each incubation was carried out in triplicate. In addition, two types of negative control samples were included: one without liver microsomes and another without the target compound. These controls were used to identify non-metabolically generated compounds and to verify the absence of interfering compounds. A positive control substrate was not included in the experimental design, as the study aimed to define the qualitative metabolic profile rather than the quantitative assessment of the microsomal activity. For all samples, 30 μL supernatant was evaporated to dryness under a gentle stream of nitrogen at 40 °C, reconstituted in 30 μL of mobile phase mixture C/D (50/50, v/v), and injected into the HPLC-QToF-MS system. No acidification step was included prior to evaporation of the pHLM incubation samples due to the small sample volume.

### High-performance liquid chromatography quadrupole time-of-flight mass spectrometry (HPLC-QToF-MS) measurements

The HPLC-QToF-MS system employed in this study consisted of an Impact II™ quadrupole time-of-flight mass spectrometer coupled to an Elute HPLC system (Bruker Daltonik, Bremen, Germany). Chromatographic separation was achieved using an Intensity Solo C18 column (100 × 2.1 mm, 1.7 μm particle size, Bruker Daltonik) equipped with a corresponding guard column (VanGuard® BEH C18 for 2.1 mm internal diameter columns). The analysis was performed using a gradient elution schedule with a total run time of 20 min. Initially, the flow rate was set at 0.2 mL/min with 4% mobile phase D for the first 0.1 min. Subsequently, mobile phase D was increased to 18.3% at 1 min. At 2.5 min, the flow rate was increased to 0.223 mL/min, and mobile phase D was raised to 50%. The gradient continued to increase linearly, reaching 99.9% mobile phase D at 14 min with a flow rate of 0.4 mL/min. These conditions were maintained until 16 min, at which point the flow rate was further increased to 0.48 mL/min. At 16.1 min, mobile phase D was decreased to 4% while maintaining the flow rate at 0.48 mL/min until 19 min. For the final minute, starting conditions were maintained for equilibration.

The column oven was maintained at 40 °C, and the autosampler was kept at 5 °C. The injection volume was 10 μL. Samples were analyzed in positive electrospray ionization (ESI +) mode, acquiring spectra in the range of *m/z* 30–650 Da at an acquisition rate of 4.0 Hz. A capillary voltage of 2500 V and an end plate offset voltage of 500 V were applied. The dry gas (nitrogen) temperature was set to 200 °C with a flow rate of 8.0 L/min, and the nebulizer gas pressure was 200 kPa. Nitrogen also served as collision gas, with a collision energy of 30 ± 9 eV. Data was acquired in both full scan/broadband collision-induced dissociation (bbCID) mode and full scan/auto-MS/MS mode, using an inclusion list for the identification of MDPiHP metabolites (Table S1, Online Resource 2). Sodium formate/acetate clusters and high-precision calibration mode were used as external and internal mass calibrations, respectively. The mass calibration was performed regularly before every batch run. Data acquisition was performed using HyStar™ version 3.2, while data processing was carried out using DataAnalysis version 4.2 (both from Bruker Daltonik). For tentative metabolite identification and characterization in both in vitro and in vivo samples, the following parameters were applied: precursor ion mass errors < 5 ppm, fragment ions mass tolerance ± 10 ppm, and a signal-to-noise ratio > 3:1.

### In silico metabolite prediction

The molecular structure of MDPiHP was represented using SMILES (Simplified Molecular Input Line Entry System), a compact text-based representation of chemical structures. SMILES strings were generated using ChemSketch (Advanced Chemistry Development, Inc.; v. 2020.1.2) and subsequently employed in three different web-based prediction tools to access potential metabolites.

Firstly, GLORYx, an open-access software developed collaboratively by the University of Vienna (Austria) and the University of Hamburg (Germany), was used to predict phase I human metabolites of MDPiHP. GLORYx assigns a prediction score to each metabolite, reflecting the estimated likelihood of its formation [16]. For further analysis, only metabolites with a prediction score of at least 20% were selected. Secondly, the EAWAG-BBD Pathway Prediction System (EAWAG-BBD/PPS) was applied to estimate microbial catabolic reactions [17]. This tool uses substructure searches, a rule-based approach, and atom-to-atom mapping to predict biotransformations. The SMILES string of MDPiHP was used along with the default parameters for aerobic transformations (six levels, ten products per level, at least three products containing a carbon atom). First-generation metabolites refer to the direct transformation products predicted from the parent compound, while second-generation metabolites represent subsequent transformation products generated from these first-generation metabolites. Thirdly, metabolite prediction was conducted using BioTransformer, which combines knowledge-based rules with machine-learning algorithms [18]. The SMILES string of the target compound was used, and the “AllHuman” transformation setting was selected to generate human-specific metabolic predictions. 

The metabolites predicted by at least one of the tools were collectively added to a HPLC-QToF/MS inclusion list (Table S1, Online Resource 2). In addition, their corresponding biotransformation pathways were included in a separate list of predicted transformations for subsequent data mining (Table S2, Online Resource 3). A review of existing literature on structurally related or analogous compounds was used to amend these lists. This multi-tool prediction strategy enabled the identification of a wide range of potential metabolites, thereby providing a more comprehensive understanding of the compound’s potential metabolic fate in humans.

## Results

### In vitro MDPiHP metabolite identification

**Fig. 1:**
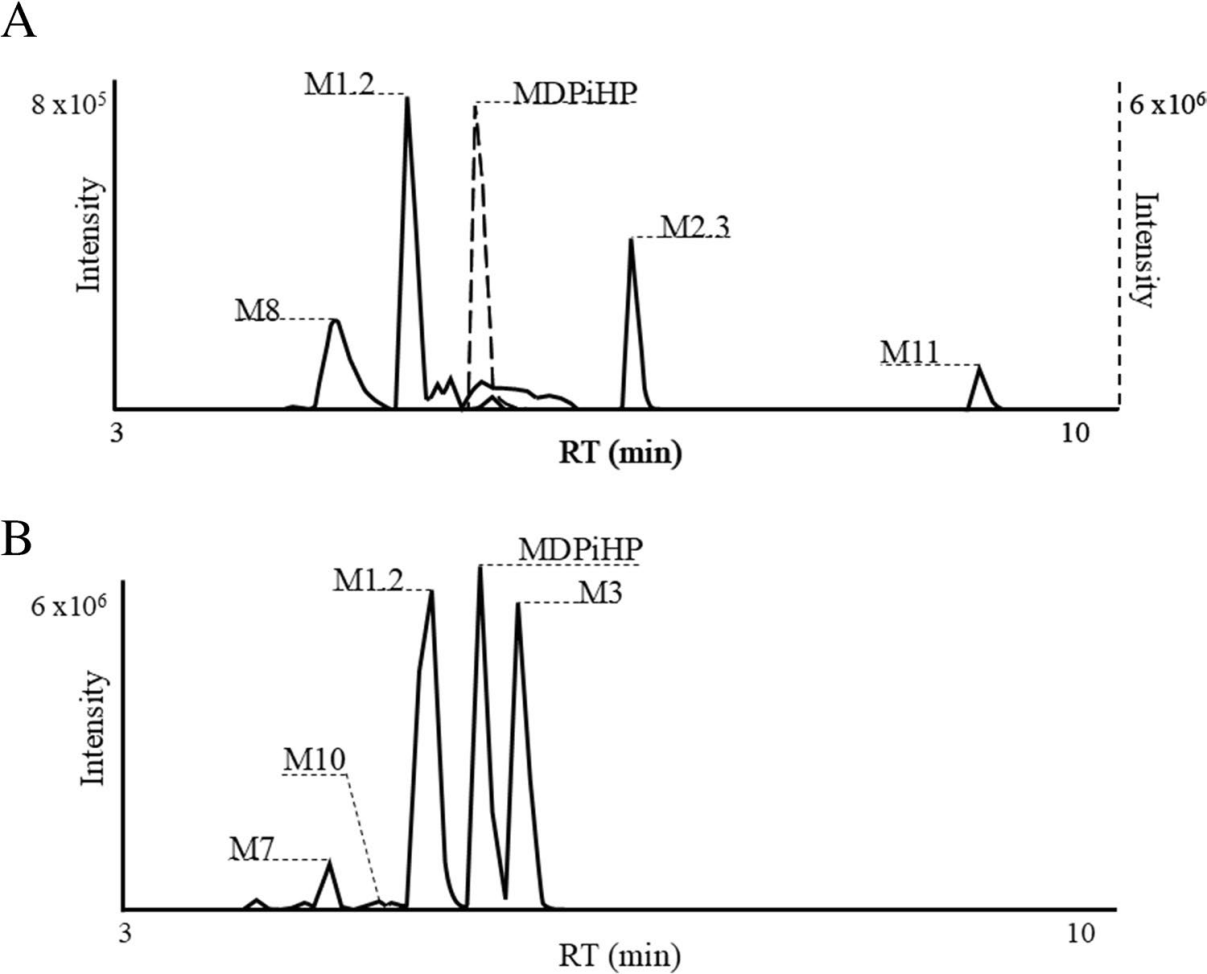
Extracted ion chromatograms of MDPiHP metabolites.** A** MDPiHP metabolites in pHLM after 2 h of incubation. Solid traces refer to the left *y*-axis, while dashed traces refer to the right *y*-axis (different units). **B** MDPiHP metabolites in urine sample U#3 as a representative of in vivo MDPiHP metabolism

After incubation of MDPiHP in pHLM, eleven metabolites were identified: M1, M1.2, M2, M2.3–M2.6, M3, M7.2, M8, and M11. Metabolite identification was performed by manual inspection of the HPLC-QToF/MS data. Specifically, extracted ion chromatograms (EIC) were generated for each expected metabolite, targeting respective precursor ions based on their predicted exact mass-to-charge ratio (*m/z*). Chromatographic peaks were identified by systematically scanning the EICs for each targeted *m/z* value. Peak identification involved a careful evaluation of peak shape, signal-to-noise ratio, and retention time consistency with respect to the parent compound. Due to their typically greater hydrophilicity compared to the respective parent compound, metabolites were consequently expected to elute earlier on the reversed-phase column. No automated data mining or metabolite identification software was used. The extracted ion chromatograms of MDPiHP and its metabolites, acquired after two hours of incubation, are presented in Fig. 1A. The most abundant metabolite tentatively identified was M8, probably resulting from demethylation of MDPiHP. M2.6 was the second most abundant metabolite, potentially formed by β-hydroxylation of the pyrrolidine ring, followed by M1.2, which might result from β-keto reduction (forming a dihydro metabolite). Minor metabolites were generated through various transformation reactions, including oxidation, hydroxylation, β-keto reduction, demethylation, methylation, and pyrrolidine ring opening followed by carboxylation. These metabolic modifications occurred both independently and in combination. Table 2 provides a summary of the proposed metabolic reactions, including the elemental compositions, calculated mass-to-charge (*m/z*) ratios, and retention times for each tentatively identified metabolite and parent MDPiHP. The ranking of metabolite abundance based on peak area is provided in Table S3 (Online Resource 4). All detected metabolites were consistently observed after 30 min, one hour, and two hours of incubation, with no substantial changes in abundance over time. In contrast, metabolites M2.1, M2.2, M4–M7.1, M9, and M10 were not detected in pHLM incubation. Specifically, chromatographic peaks corresponding to these metabolites were neither observed in the incubated samples nor in the blank control incubations under the applied analytical conditions.

**Table 2:** Proposed metabolic reaction, elemental composition, accurate mass of the molecular ion, and retention time. The rank was determined based on the peak area of the protonated molecules

ID	Proposed metabolic reaction	Elemental composition	[M+H]^+^	RT (min)	Average rank			
					pHLM	U	FB	S&P
-	MDPiHP	C_17_H_24_NO_3_	290.1751	5.6	-	-	-	-
M1.2	β-Keto reduction (Dihydro-MDPiHP)	C_17_H_26_NO_3_	292.1907	5.2	3	1	1	1
M2.3	β-Hydroxylation on pyrrolidine ring	C_17_H_24_NO_4_	306.1700	5.7	2	ND	ND	ND
M3	α-Hydroxylation + pyrrolidine ring opening + carboxylation	C_17_H_24_NO_5_	322.1649	5.8	PD	2	2	2
M7	β-Keto reduction + aliphatic hydroxylation	C_17_H_26_NO_4_	308.1856	4.5	ND	3	PD	PD
M8	*O*-Demethylenation	C_16_H_24_NO_3_	278.1751	4.6	1	PD	ND	PD
M10	*O*-Demethylenation + *O*-methylation	C_17_H_25_NO_3_	292.1907	4.8	ND	4	3	3
M11	β-Oxidation on pyrrolidine ring	C_17_H_22_NO_4_	304.1543	9.4	4	PD	ND	ND

*FB*, femoral blood; *ND*, not detected; *P*, plasma; *PD*, partially detected; *pHLM*, pooled human liver microsomes; *RT*, retention time; *S*, serum; *U*, urine

### Human samples

A total of eleven samples analyzed for MDPiHP, including three femoral blood samples, four serum samples, one plasma sample, and three urine samples. Measured concentrations ranged from approx. 1.5 to approx. 34 ng/mL across the analyzed samples. In addition, MDPHP—the positional isomer of MDPiHP—was also detected in urine specimens #1 and #3. Although chromatographic resolution of the two compounds was sufficient to enable reliable identification (see also “Quantification of MDPiHP by LC–MS/MS” and Figure S1, Online Resource 1), accurate quantification was not possible when both analytes were present. Consequently, in these cases MDPiHP was reported as “qualitatively detected”. The full concentration data are presented in Table S4 (Online Resource 5).

#### In vivo MDPiHP metabolite identification

Twenty-two metabolites (M1–M2.2, M2.4, M3–M11) were detected across human urine, femoral blood, serum, and plasma specimens. In urine samples, the predominant metabolite was M1.2 (probably β-keto reduction, forming dihydro-MDPiHP), followed in abundance by M3, which might be formed via α-hydroxylation, subsequent pyrrolidine ring opening, and oxidation to the corresponding carboxylic acid. M7, a metabolite resulting from a combination of β-keto reduction and hydroxylation, was also detected at high abundance. Similarly, M10, potentially formed through consecutive *O*-demethylenation (ring opening) and *O*-methylation, was detected in notable amounts.

**Fig. 2:**
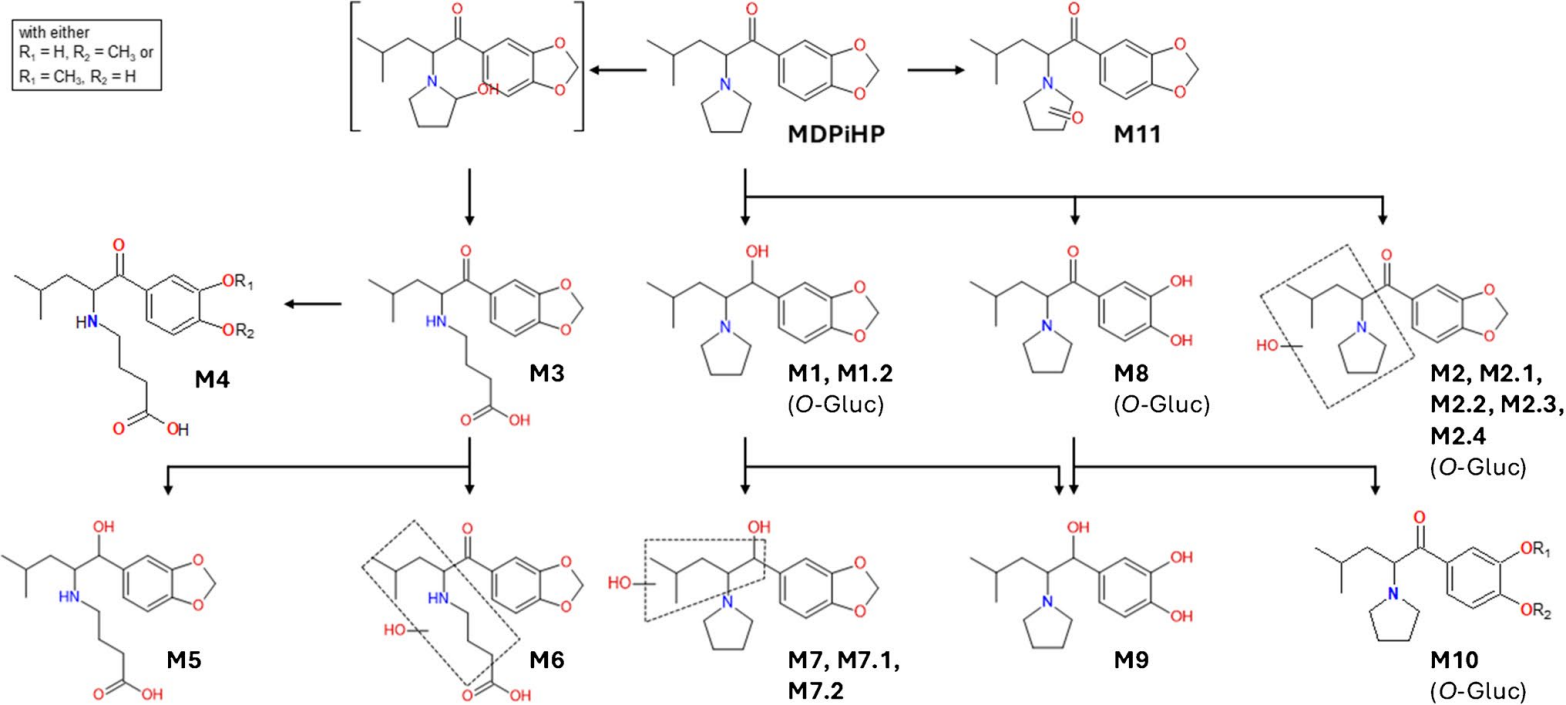
MDPiHP proposed in vivo metabolic pathway. The structures of M4 and M10 incorporate aromatic substituents OR_1_ and OR_2_, with the constraint that either R_1_ = CH_3_ and R_2_ = H, or R_1_ = H and R_2_ = CH_3_.

The metabolite profile observed in femoral blood closely resembled that found in urine samples. However, M7 was found exclusively in the femoral blood sample of case #1. In contrast, serum and plasma samples both showed comparable patterns of metabolic distribution, although with slight differences in the relative abundance of individual compounds. In both matrices, the parent compound MDPiHP exhibited the highest abundance, followed by M1.2, M3, and M10. Notably, among serum samples, M7 was detected only in serum #4. Minor metabolites were possibly formed through a diverse range of biotransformations, including oxidation, reduction, hydroxylation, demethylation, methylation, and pyrrolidine ring opening followed by oxidation to the corresponding carboxylic acid. Additional transformations, such as nucleophile-driven cyclization and dehydration, were also observed, either as standalone processes or in combination with others. Tables 2 and S4 summarize the proposed metabolic pathways, including molecular formulas, calculated *m/z* values, and retention times of MDPiHP and its metabolites. Notably, M2.5 and M2.6 were not detected in any of the analyzed human specimens. Figure 1B presents the extracted ion chromatograms of MDPiHP and its metabolites in urine sample #3, which was selected as a representative example of human metabolite profiles. The proposed metabolic pathway is shown in Fig. 2.

### Fragmentation spectra of MDPiHP and in vivo major metabolites

**Fig. 3:**
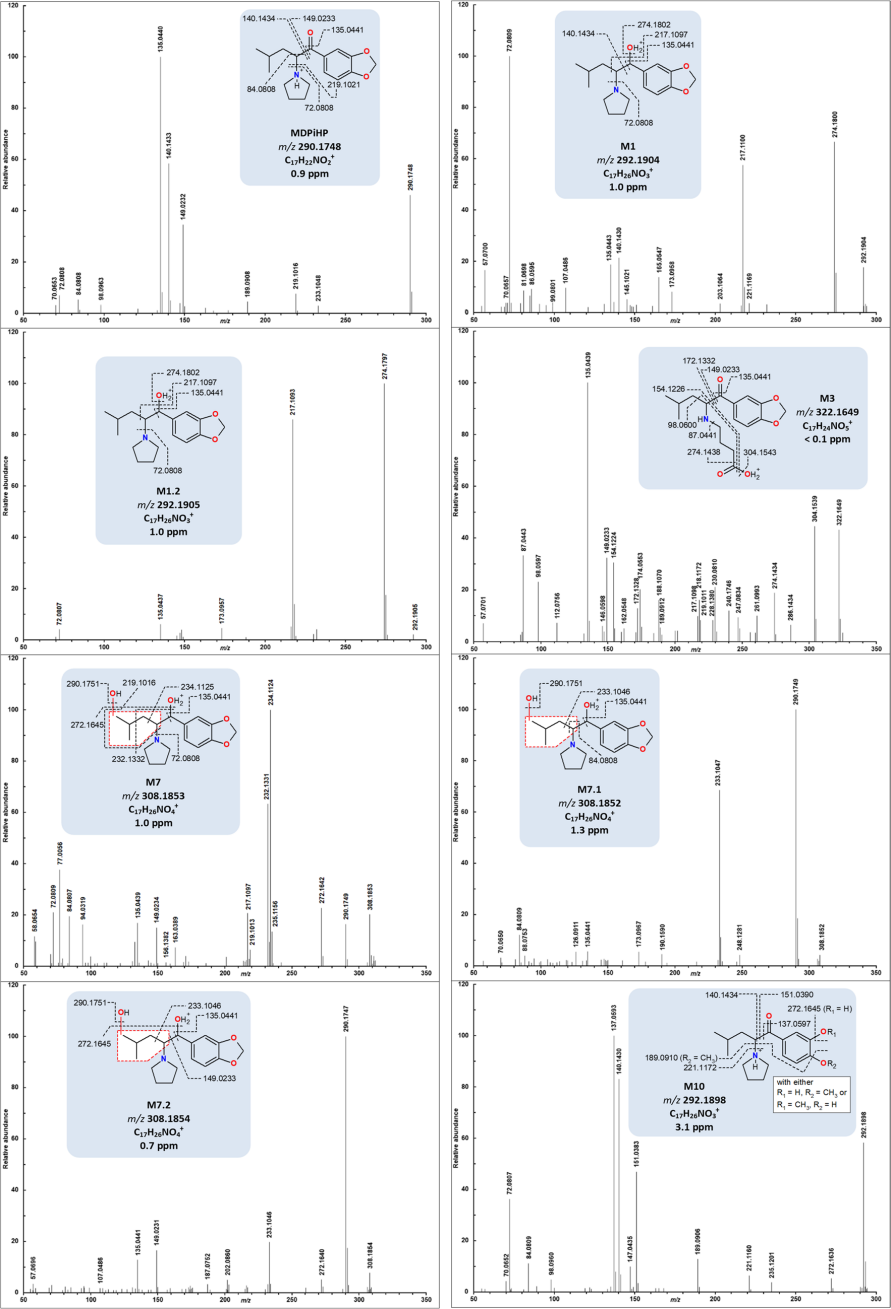
Fragmentation spectra of MDPiHP and its major in vivo metabolites obtained by LC–QToF/MS. The structure of metabolite M10 contains aromatic substituents OR_1_ and OR_2_, where either R_1_ = CH_3_ and R_2_ = H, or R_1_ = H and R_2_ = CH_3_. Dashed lines in the molecular structures represent simplified fragmentation pathways and the corresponding fragment masses; detailed schemes including potential mass spectrometric rearrangements are provided in Figs. S3–S7 (Online Resources 7–11) in the Supplementary Material

To tentatively elucidate the structures of MDPiHP metabolites, the fragmentation behavior of the parent compound was first examined. This approach is important because many metabolic transformations potentially involve the introduction or modification of functional groups while the main molecular framework remains unchanged. When fragment ions containing these functional groups appear identical to fragments in the parent compound, it is possible to deduce the most likely site of biotransformation. The fragmentation spectra of MDPiHP and its major in vivo metabolites are shown in Fig. 3. The signals were acquired in positive ionization mode. The spectra of minor in vivo metabolites are shown in Figure S2 (Online Resource 6).

#### MDPiHP

The protonated parent compound MDPiHP ([M+H]^+^, *m/z* 290.1748, C_17_H_24_NO_3_^+^, 0.9 ppm) eluted at 5.6 min. Its spectrum featured prominent fragment ions at *m/z* 140.1433 (C_9_H_18_N^+^, 0.5 ppm), consistent with the loss of the piperonal moiety. Another key fragment at *m/z* 149.0232 (C_8_H_5_O_3_^+^, 0.8 ppm), is likely formed through an alpha-cleavage. The loss of the pyrrolidine ring from the protonated parent compound could generate the fragment at *m/z* 219.1016 (C_13_H_15_O_3_^+^, −0.1 ppm). Subsequently, a loss of isobutene via a McLafferty-type rearrangement could yield the minor fragment at *m/z* 163.0392 (C_9_H_7_O_3_^+^, −1.2 ppm) [19]. Furthermore, a possible Wagner-Meerwein-type rearrangement and a carbon monoxide loss could result in the fragment at *m/z* 135.0440 (C_8_H_7_O_2_^+^, 0.4 ppm) [20, 21]. The ions at *m/z* 84.0808 (C_5_H_10_N^+^, −0.3 ppm) and *m/z* 72.0808 (C_4_H_10_N^+^, −0.3 ppm) could represent characteristic fragments derived from the pyrrolidine moiety, such as protonated pyrrolidine (for *m/z* 72.0808) and related iminium ions. The proposed fragmentation pathway is depicted in Figure S3 (Online Resource 7). In summary, these features facilitated the elucidation of MDPiHP metabolite structures.

#### Metabolites M1 and M1.2

Metabolites M1 and M1.2 ([M+H]^+^, *m/z* 292.1905, C_17_H_26_NO_3_^+^, 0.7 ppm) eluted at 5.5 and 5.2 min, respectively. Both metabolites share the same fragments, indicating that they could share closely related chemical structures. The fragmentation pattern displayed the most intense fragment at *m/z* 274.1800 (C_17_H_24_NO_2_^+^, 0.7 ppm), which might correspond to a water loss. A subsequent homolytic cleavage of the aliphatic chain could lead to the radical ion at *m/z* 217.1093 (C_13_H_15_NO_2_^+•^, 1.8 ppm). Considering an alternative protomer of the protonated M1/M1.2, with protonation occurring at the pyrrolidine ring instead of the hydroxy group, the loss of the pyrrolidine ring could generate *m/z* 221.1178 (C_13_H_17_O_3_^+^, 2.9 ppm). Analogous to the parent compound, the ion at *m/z* 165.0546 (C_9_H_9_O_3_^+^, −0.6 ppm) could be the result of an isobutene loss, while the ion at *m/z* 135.0443 (C_8_H_7_O_2_^+^, −1.5 ppm) could be subsequently formed through a Wagner-Meerwein-type rearrangement followed by a formaldehyde elimination. Furthermore, the fragments at *m/z* 140.1430 (C_9_H_18_N^+^, 2.9 ppm) and *m/z* 72.0807 (C_4_H_10_N^+^, < 0.1 ppm) might correspond to the aliphatic chain with the pyrrolidine moiety and the isolated pyrrolidine ring, respectively. Taken together, these fragment ions, especially the observed water loss, are consistent with a dihydro-MDPiHP metabolite. Since reduction of the keto group would introduce a second stereocenter, M1 and M1.2 might represent diastereomers. The proposed fragmentation pathway is depicted in Figure S4 (Online Resource 8).

#### Metabolite M3

Metabolite M3 ([M+H]^+^, *m/z* 322.1649, C_17_H_24_NO_5_^+^, < 0.1 ppm) eluted at 5.8 min. The fragment ion at *m/z* 304.1539 (C_17_H_22_NO_4_^+^, 1.1 ppm) could correspond to the loss of a water molecule. A subsequent loss of formaldehyde could produce the ion at *m/z* 274.1434 (C_16_H_20_NO_3_^+^, 1.1 ppm). A further loss of C_3_H_5_N, potentially reflecting the formation of an azacycle via nucleophilic attack of the nitrogen atom at the allylic carbon atom, might generate the ion at *m/z* 219.1011 (C_13_H_15_O_3_^+^, 3.2 ppm). From the ion at *m/z* 219.1011, the fragmentation appears to proceed along a pathway similar to that of the parent compound, leading to ions at *m/z* 149.0233 (C_8_H_5_O_3_^+^, 0.1 ppm) and *m/z* 135.0439 (C_8_H_7_O_2_^+^, 1.5 ppm). Alternatively, considering a potential alternative protomer of the protonated M3, in which protonation might occur at the amine, the loss of the piperonal moiety could result in the fragment ion at *m/z* 172.1328 (C_9_H_18_NO_2_^+^, 2.3 ppm). The loss of the isopentyl imine chain could then yield the ion at *m/z* 87.0443 (C_4_H_7_O_2_^+^, −2.3 ppm). Alternatively, a subsequent water loss could also yield a fragment at *m/z* 154.1224 (C_9_H_16_NO^+^, 1.3 ppm), while a further loss of isobutene potentially results in the ion at *m/z* 98.0597 (C_5_H_8_NO^+^, 3.1 ppm). Overall, this tentative fragmentation pathway could be consistent with a ring-opened metabolite that has undergone further oxidation to the corresponding terminal carboxylic acid. The proposed fragmentation pathway is depicted in Figure S5 (Online Resource 9).

#### Metabolite M7

Metabolite M7 ([M+H]^+^, *m/z* 308.1853, C_17_H_26_NO_4_^+^, 1.0 ppm) eluted at 4.5 min. The fragment ion at *m/z* 290.1749 (C_17_H_24_NO_3_^+^, 0.7 ppm) could indicate a water loss. Subsequently, fragmentation might involve the cleavage of C_3_H_6_O, which—depending on the site of hydroxylation—could correspond to either the loss of acetone or propanal, yielding an ion at *m/z* 232.1331 (C_14_H_18_NO_2_^+^, 0.4 ppm). Alternatively, a second water loss could form the ion observed at *m/z* 272.1642 (C_17_H_22_NO_2_^+^, 1.1 ppm). A possible sequential homolytic cleavage of an isobutene radical might generate the radical ion at *m/z* 217.1097 (C_13_H_15_NO_2_^+•^, < 0.1 ppm). Starting instead from an alternative protomer of the protonated M7, a subsequent water loss (producing *m/z* 290.1749), could lead to the predominant fragment at *m/z* 234.1124 (C_13_H_16_NO_3_^+^, 0.4 ppm), which may correspond to the cleavage of isobutene. Another possible fragmentation pathway could involve the loss of a pyrrolidine ring, yielding the ion at *m/z* 219.1013 (C_13_H_15_O_3_^+^, 1.4 ppm). The subsequent fragmentation originating from *m/z* 219.1011 seems to follow a pathway comparable to that proposed for the parent compound. The fragment ions at *m/z* 72.0809 (C_4_H_10_N^+^, −1.4 ppm) and *m/z* 84.0807 (C_5_H_10_N^+^, 1.2 ppm) might correspond to the pyrrolidine ring moiety. Taken together, these fragment ions, particularly the observed consecutive water losses, are consistent with a potentially hydroxylated dihydro-MDPiHP metabolite. In line with M1 and its isomers, the reduction of the keto group, combined with hydroxylation at the alkyl chain, would introduce two additional stereocenters. Therefore, M7, M7.1 and M7.2 could represent diastereomers and/or positional isomers. However, it is possible that the non-detectability of the theoretically calculated diastereomers may result from a potential coelution or low abundance. The proposed fragmentation pathway is depicted in Figure S6 (Online Resource 10).

#### Metabolite M10

Metabolite M10 ([M+H]^+^, *m/z* 292.1898, C_17_H_26_NO_3_^+^, 3.1 ppm) eluted at 4.8 min. A potential loss of the pyrrolidine ring might yield the fragment at *m/z* 221.1160 (C_13_H_17_O_3_^+^, 5.4 ppm). The possible cleavage of the alkyl chain could generate the fragment ion at *m/z* 151.0383 (C_8_H_7_O_3_^+^, 4.6 ppm). In analogy to the parent, a pathway involving two rearrangements might also occur. Specifically, a potential McLafferty-type rearrangement could yield *m/z* 165.0435 (C_9_H_9_O_3_^+^, 1.8 ppm). Subsequently, a possible Wagner-Meerwein-type rearrangement might result in the fragment ion at *m/z* 137.0593 (C_8_H_9_O_2_^+^, 2.9 ppm). This latter ion could suggest the presence of a methoxyphenol moiety. A minor pathway might start from an alternative protomer of the protonated M10. In this case, a possible water loss combined with an extension of the aromatic system could lead to *m/z* 272.1636 (C_17_H_22_NO_2_^+^, 3.3 ppm). A subsequent cleavage of the pyrrolidine ring and a potential loss of the isobutene unit might yield *m/z* 147.0435 (C_9_H_7_O_2_^+^, 4.1 ppm). The ions at *m/z* 140.1430 (C_9_H_18_N^+^, 2.9 ppm), *m/z* 72.0807 (C_4_H_10_N^+^, 1.4 ppm), and *m/z* 84.0809 (C_5_H_10_N^+^, −1.2 ppm) could correspond to fragments derived from the pyrrolidine ring, as described in “MDPiHP”. The proposed fragmentation pathway is depicted in Figure S7 (Online Resource 11).

### In silico predicted metabolites

In silico predictions of metabolite formation were evaluated using three freeware tools [16–18]. A total of 23 putative metabolites were generated, including 21 phase I and two phase II metabolites. Of these, 15 metabolites were predicted by more than one tool. In particular, GLORYx generated ten putative phase I metabolites (pG1 to pG10, ranked by descending prediction score). EAWAG-BBD also predicted ten phase I metabolites, including four first-generation (from pE1 to pE4) and six second-generation metabolites (pEn.1 to pEn.6). According to the software’s scoring system, eight out of these ten metabolites were considered more likely to occur than the remaining candidates. BioTransformer predicted a single phase I metabolite (pBT1) and two phase II metabolites (pBT1.1 and pBT1.2). However, prediction scores were not provided. The most common postulated biotransformations were hydroxylation, *O-*dealkylation, *N-*dealkylation, and reduction. The biotransformations of the predicted metabolites are listed in Table 2.

## Discussion

### MDPiHP major in vivo metabolites

Consistent with the previous literature, our study detected metabolites whose potential biotransformations are characteristic of structural analogs of MDPiHP [9–11, 22, 23]. Notably, Meyer et al. [21] and Grap et al. [9, 22] reported demethylenyl-methyl metabolites as the predominant urinary metabolites of MDPV and MDPHP. These findings suggest *O*-methylation constitutes a major metabolic pathway shared across related analogs and could serve as a potential biomarker. In our study, metabolite M10 was found abundantly in its free form, with only minor levels of glucuronide conjugates observed. The formation of M10 could occur through CYP2D6-mediated *O-*demethylenation of the 3,4-methylenedioxy ring to a catechol intermediate, followed by catechol-*O*-methyltransferase (COMT)-mediated *O-*methylation. Similar biotransformations have been documented for MDMA and related 3,4-methylenedioxyphenyl analogs in vivo [22–24]. The fragment ions at *m/z* 137.0593 and *m/z* 151.0383 support the postulated dioxole ring opening. The opening of the dioxole ring may occur via an oxidative mechanism at the methylenedioxy group, primarily catalyzed by CYP2D6, with additional contributions from other CYP450 isoenzymes [22, 24]. These metabolites can be relevant for toxicological assessment as they may exhibit differing activity at human monoamine transporters [25, 26].

Interestingly, M1 and M1.2 share the same molecular mass and formula as M10, but their fragmentation patterns differ, indicating different chemical structures. The fragment ions of M1 and M1.2 at *m/z* 221.117 and *m/z* 165.0546 could indicate the presence of a hydroxy group and an unchanged 3,4-methylenedioxy-substituted benzene ring, implying β-keto reduction of the carbonyl group as the key biotransformation. This reduction likely produces two stereocenters, yielding two distinguishable diastereomers, M1 and M1.2. β-Keto reduction is a common biotransformation among synthetic cathinones (forming dihydro metabolites), as their carbonyl groups can be reduced in vivo to secondary alcohols by reductases [27]. While previous in vivo metabolism studies of MDPHP reported only low or negligible levels of dihydro metabolites [9–11], our investigation revealed a notably high abundance of M1.2, primarily in its free form, emphasizing its potential as a biomarker of MDPiHP intake.

The hydroxylation of M1/M1.2 could potentially lead to the hydroxylated dihydro metabolite M7, as well as its postulated positional isomers or diastereomers M7.1 and M7.2. The presumably intact dioxole ring in M7 suggests that hydroxylation occurred at the alkyl side chain rather than at the aromatic ring. However, the exact position of the additional hydroxy group cannot be unambiguously assigned based on the fragmentation spectra. Interestingly, Kavanagh et al. and Grap et al. did not report the corresponding metabolites in their MDPHP metabolism studies [9, 11]. Instead, they described a hydroxylated metabolite featuring an open dioxole ring, which nevertheless shared the same molecular formula and mass as M7 identified in our study. In our case, the structural interpretation of M7 was supported by fragment ions at *m/z* 290.1749 and *m/z* 272.1642. These ions, potentially corresponding to two consecutive losses of water, point to the presence of two hydroxy groups while maintaining an intact dioxole ring. It appears unlikely that both hydroxy groups are attached to the same carbon atom, since such configuration would create an unstable intermediate that would readily eliminate water to stabilize. Moreover, all proposed fragment structures display an unchanged pyrrolidine ring, suggesting that no hydroxylation occurred at this site. Consequently, only one of the remaining aliphatic positions can reasonably accommodate the second hydroxy group.

Metabolite M3 was among the major metabolites detected. It might arise through hydroxylation at the alpha position of the pyrrolidine ring, thereby forming a labile hemiaminal. The intermediate could subsequently undergo ring opening, followed either by reduction to a terminal alcohol (not observed) or oxidation to the corresponding carboxylic acid moiety (M3). The fragmentation spectrum of M3 showed close similarity to those reported for the respective MDPHP and MDPV metabolites, which have previously been identified as major biomarkers of consumption [9, 11, 22, 23].

A key limitation of cathinone metabolism studies is the high instability of these compounds in biological matrices, including blood and urine, due to thermal, pH-dependent, and enzymatic degradation. However, synthetic cathinones possessing 3,4-methylenedioxy or *N*-pyrrolidine rings, such as MDPV or MDPiHP, show lower degradation rates in human samples compared to other substituted cathinones [28, 29]. Degradation products often include dihydro (reduced β-keto) metabolites. This may lead to lower concentrations of the parent drug and complicates the interpretation of metabolite ratios and degradation kinetics, especially in post-mortem toxicology. In addition, an underestimation of parent drug concentrations potentially leads to incorrect toxicological assessments.

Furthermore, stereoselective metabolism and related toxicity should be taken into consideration. Enantiomers of cathinone-type drugs and their metabolites can exhibit different cytotoxicity and hepatotoxicity. Therefore, chiral discrimination with regard to metabolic enzymes may impact overall pharmacokinetics and toxicodynamics [30–32]. Variability in human cytochrome P450 isoenzymes, especially CYP2D6 and COMT polymorphisms, contributes to interindividual differences in metabolite profiles, which presents challenges for biomarker selection. Standard protocols to inhibit enzymatic degradation include the addition of sodium fluoride to blood samples, which stabilizes analytes at room temperature. Nevertheless, freezing samples at −40 °C or lower remains essential for long-term storage [33].

An additional point requiring consideration is the co-presence of the positional isomer MDPHP in urine samples U#1 and U#3. MDPHP and MDPiHP share a high degree of structural similarity and are known to undergo overlapping biotransformation pathways [9–11, 22, 23]. Consequently, some metabolites potentially coelute under the applied chromatographic conditions. The chromatographic resolution of all metabolites originating from both analytes cannot be unequivocally determined. This limitation is particularly relevant for urine samples U#1 and U#3, which were the only samples in which certain metabolites (e.g., M2.4, M2 *O-*gluc, M5, M6, M9, M10.1 *O-*gluc, and M11) were detected. Therefore, a partial contribution of MDPHP to the formation of these metabolites cannot be completely excluded. Nevertheless, metabolite assignment was supported by high-resolution mass spectrometry, characteristic fragmentation patterns, and consistency with a previously reported in vivo metabolism study of MDPHP and related 3,4-methylenedioxy-substituted cathinones [9–11, 22, 23].

Based on comprehensive metabolic profiling, we propose metabolites M1.2, M3, and M10, together with parent MDPiHP, as reliable biomarkers of MDPiHP intake in urine, blood, serum, and plasma samples. Since dihydro metabolites are known for their stability in whole blood samples providing longer detection windows, M1.2 might also be promising for identifying past intake. Notably, metabolite M7 appeared consistently in urine samples, but only occasionally in other matrices. This observation suggests M7 as a potential urine-specific biomarker.

### Comparison of in vitro and in vivo metabolism

Substantial differences were observed between the major metabolites identified in biological samples and those detected in pHLM incubation (Table 2). Notably, metabolites M7 and M10 were not detected in pHLM at any time point. In contrast, the major in vitro metabolites were either absent or only partially detected in human samples. To facilitate an intuitive comparison between in vitro and in vivo metabolism, metabolites were ranked according to their relative signal intensity within each matrix. This ranking was intended to reflect metabolite predominance under the respective experimental conditions and to support a qualitative comparison across different matrices.

These discrepancies might reflect the distinct metabolic environments present in vivo and in vitro. Although pHLM are widely employed as a model to approximate in vivo metabolism, they provide only a limited set of enzymes and isoenzymes, and lack the complexity of the physiological milieu. In such incubations, the major biotransformations are primarily mediated by cytochrome P450 enzymes and isoenzymes, which could involve reactions such as hydroxylation, *O-*dealkylation, and β-keto reduction [22, 27]. Consistent with this enzymatic repertoire, the most prominent in vitro metabolites were M1.2, M2.3, and M8. In contrast, the in vivo metabolic environment supports more diverse, sequential, and combined transformations. Therefore, M7, M10, and M3 were the most abundant metabolites detected in biological samples.

In addition to the biological differences, methodological aspects may also contribute to the observed divergence in metabolite profiles. The human samples were processed using a solid-phase extraction approach, which may result in incomplete recovery of highly polar metabolites, whereas pHLM incubation samples were precipitated with acetonitrile, optimized for broader analyte coverage. Consequently, differences in relative signal intensities and abundance ranking may partly reflect extraction-related biases, and comparisons based on peak areas should be interpreted within the context of each matrix.

In this study, the metabolic profile generated in pHLM incubations did not fully reflect the in vivo results, which may be attributed to the inherent limitations of the applied in vitro model. While pHLM assays represent an established tool for investigating hepatic metabolism, their predictive accuracy can vary depending on the compound and the metabolic pathways involved. Under physiological conditions, metabolites may undergo processes such as enterohepatic circulation or active transport, potentially facilitating rapid phase II biotransformations to promote elimination [34]. This mechanism might explain the discrepancy observed for M8. In vitro*,* M8 likely formed through *O-*demethylenation, and emerged as a major metabolite. However, in vivo, M8 was detected only at low levels and with limited prevalence. Such a difference could indicate that M8 is rapidly converted into phase II metabolites, potentially yielding derivatives such as M10 and M7, which were abundant in biological samples. Consequently, the enzyme composition used, absence of complete phase II processes, and the reduced environmental complexity of the in vitro system likely contribute to the divergent metabolite profiles observed between in vitro and in vivo conditions. The observed differences between in vitro and in vivo profiles highlight the complementary nature of these experimental approaches and underscore the need to interpret pHLM data as part of an integrated metabolic assessment rather than as a standalone predictor of in vivo outcomes.

## Conclusion

This study represents the first comprehensive investigation into the metabolism of MDPiHP, a synthetic cathinone whose rapid emergence in Europe has raised substantial public health concerns. Using high-resolution mass spectrometry to analyze biological samples, we identified the parent compound MDPiHP along with metabolites M1.2, M3, and M10 as biomarkers of intake in blood, serum, plasma, and urine. Notably, metabolite M7 could serve as a urine-specific biomarker. Comparative in vitro experiments using pooled human liver microsomes revealed metabolic patterns that differ from those observed in vivo, highlighting the limitations of in vitro assays in capturing the full complexity of human metabolism. A key limitation in interpreting drug concentrations in case work is the known pronounced instability of cathinones in biological matrices, which can lead to lower concentrations of the parent drug and shifts in metabolite ratios. Finally, stereoselective metabolism and resulting toxicity remain an important unexplored area. Taken together, our findings provide a fundamental metabolic profile of MDPiHP, which forms an important basis for forensic and clinical toxicology and paves the way for future studies on its detection, toxicity, and pharmacokinetics.

## Supplementary Information

Below is the link to the electronic supplementary material.

Supplementary file1 (PDF 1.29 MB)

## Data Availability

All data generated or analyzed during this study are included in the published article and in the Supplementary files.
