# Supplementary material for: Comprehensive metabolic profiling of the new designer stimulant MDPiHP—in vitro and in vivo identification of potential biomarkers for detection in human samples
Source: Anal Bioanal Chem. 2026 Mar 12;418(9):2865–78. doi: 10.1007/s00216-026-06417-1 (PMC13079469; doi:10.1007/s00216-026-06417-1)
Supplement: Supplementary file 1 — Supplementary file1 (PDF 1.29 MB) [file 216_2026_6417_MOESM1_ESM.pdf]

Comprehensive Metabolic Profiling of the New Designer Stimulant MDPiHP - *In Vitro* and *In Vivo* Identification of Potential Biomarkers for Detection in Human Samples

Analytical and Bioanalytical Chemistry

Aurora Balloni[1,2,†] · Johannes Kutzler[1,3,†] · Giuseppe Basile[2] · Francesco P. Busardò[2] · Jeremy Carlier[2] · Volker Auwärter[1]

[1]Institute of Forensic Medicine, Forensic Toxicology, Medical Center – University of Freiburg, Faculty of Medicine, University of Freiburg, Germany

[2]Marche Polytechnic University, Department of Biomedical Sciences and Public Health, Section of Legal Medicine, Unit of Forensic Toxicology, Ancona, Italy

[3]Hermann Staudinger Graduate School, University of Freiburg, Germany

[†]Both authors contributed equally to the manuscript.

Correspondence

Prof. Dr. Volker Auwärter

Albertstr. 9, 79104 Freiburg, Germany

E-mail: volker.auwaerter@uniklinik-freiburg.de

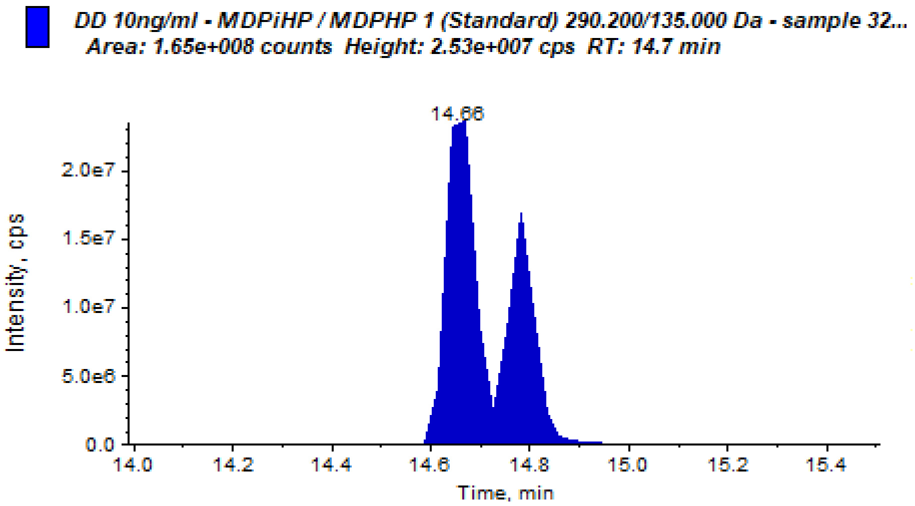

Fig S1 LC-QToF/MS chromatograms of reference standard solutions of MDPiHP and MDPHP (10 ng/mL each). The peak resolution between MDPiHP and MDPHP was 1.14, corresponding to a retention time difference of 0.12 min, which was sufficient for reliable peak identification. The retention times were 14.66 min for MDPiHP and 14.80 min for MDPHP.

Table S1. Inclusion list used during liquid chromatography-quadrupole time-of-flight mass spectrometry (HPLC-QToF-MS) for MDPiHP metabolites identification

3,4-Methylenedioxy-α-Pyrrolidinoisohexanophenone (MDPiHP)

**Transformation**	**Elemental composition**	**$[M+H]^+$ *m/z***	**$[M-H]^-$ *m/z***
Parent (MDPiHP)	$C_{17}H_{23}NO_3$	290.1751	288.1605
+2O	$C_{17}H_{23}NO_5$	322.1648	320.1503
+6C +10H +6O	$C_{23}H_{33}NO_9$	468.2228	466.2082
+2H +2O	$C_{17}H_{25}NO_5$	324.1805	322.1659
–1C	$C_{16}H_{23}NO_3$	278.1750	276.1605
+2H	$C_{17}H_{25}NO_3$	292.1907	290.1761
+5C +8H +6O	$C_{22}H_{31}NO_9$	454.2071	452.1926
–2H +2O	$C_{17}H_{21}NO_5$	320.1492	318.1346
+6C +10H +7O	$C_{23}H_{33}NO_{10}$	484.2177	482.2031
+6C +8H +8O	$C_{23}H_{31}NO_{11}$	498.1960	496.1824
+1O	$C_{17}H_{23}NO_4$	306.1699	304.1554
+6C +8H +7O	$C_{23}H_{31}NO_{10}$	482.2020	480.1875
–1C +1O	$C_{16}H_{23}NO_4$	294.1699	292.1554
+2H +1O	$C_{17}H_{25}NO_4$	308.1856	306.1710
–2H +1O	$C_{17}H_{21}NO_4$	304.1543	302.1397
–1C +2O	$C_{16}H_{23}NO_5$	310.1648	308.1503
+6C +10H +8O	$C_{23}H_{33}NO_{11}$	500.2126	498.1980
+2H +5O +1S	$C_{17}H_{25}NO_8S$	404.1373	402.1228
+3O	$C_{17}H_{23}NO_6$	338.1598	336.1452
–1C –2H +1O	$C_{16}H_{21}NO_4$	292.1543	290.1397
–4C –4H	$C_{13}H_{19}NO_3$	238.1437	236.1292
–5C –6H	$C_{12}H_{17}NO_3$	224.1281	222.1135
+2H +3O +1S	$C_{17}H_{25}NO_6S$	372.1475	370.1329
+5C +8H +7O	$C_{22}H_{31}NO_{10}$	470.2020	468.1875
–1C +2H	$C_{16}H_{25}NO_3$	280.1907	278.1761
+5C +10H +6O	$C_{22}H_{33}NO_9$	456.2228	454.2082
–13C –14H –3O	C_4H_9N	72.0807	70.0662
–13C –14H –2O	C_4H_9NO	88.0756	86.0611
–4C –9H +1O	$C_{13}H_{14}O_4$	235.0964	233.0819
–4C –9H +2O	$C_{13}H_{14}O_5$	251.0914	249.0768
–2H	$C_{17}H_{21}NO_3$	288.1594	286.1448

Table S2. 3,4-Methylenedioxy-α-Pyrrolidinoisohexanophenone (MDPiHP) putative metabolites predicted with GLORYx, EAWAG-BBP and BioTransformer freeware and their prediction score.

3,4-Methylenedioxy-α-Pyrrolidinoisohexanophenone (MDPiHP)

**ID**	**Transformation**	**Elemental composition**	**Score**	**Simplified molecular-input line-entry system (SMILES)**	**Comment**
pG1	Aliphatic hydroxylation	$C_{17}H_{23}NO_4$	78.0%	CC(C)CC(C(=O)c1ccc2c(c1)OC(O)O2)N1CCCC1	=pG2, pG5, pG8, pG10, pE4
pG2	α-Hydroxylation + dioxolane ring opening	$C_{17}H_{23}NO_4$	78.0%	CC(C)CC(C(=O)c1ccc(OC=O)c(O)c1)N1CCCC1	=pG1, pG5, pG8, pG10, pE4
pG3	Demethylation + *O*-methylation	$C_{17}H_{25}NO_3$	78.0%	COc1ccc(C(=O)C(CC(C)C)N2CCCC2)cc1O	=pG7, pBT1
pG4	O-Dealkylation of methylenedioxyphenyl	CHO_2	78.0%	O=C[O-]	
pG5	α-Hydroxylation + dioxolane ring opening	$C_{17}H_{23}NO_4$	78.0%	CC(C)CC(C(=O)c1ccc(O)c(OC=O)c1)N1CCCC1	=pG1, pG2, pG8, pG10, pE4
pG6	Demethylation	$C_{16}H_{23}NO_3$	78.0%	CC(C)CC(C(=O)c1ccc(O)c(O)c1)N1CCCC1	
pG7	Demethylation + *O*-methylation	$C_{17}H_{25}NO_3$	78.0%	COc1cc(C(=O)C(CC(C)C)N2CCCC2)ccc1O	=pG3, pBT1
pG8	Aliphatic hydroxylation	$C_{17}H_{23}NO_4$	34.4%	CC(C)(O)CC(C(=O)c1ccc2c(c1)OCO2)N1CCCC1	=pG1, pG2, pG5, pG10, pE4
pG9	Carboxylation	$C_{17}H_{21}NO_5$	34.4%	CC(CC(C(=O)c1ccc2c(c1)OCO2)N1CCCC1)C(=O)O	
pG10	Aliphatic hydroxylation	$C_{17}H_{23}NO_4$	34.4%	CC(CO)CC(C(=O)c1ccc2c(c1)OCO2)N1CCCC1	=pG1, pG2, pG5, pG8, pE4
pE1	Alkyl hydroxylation	$C_{17}H_{23}NO_3$	L	CC(C)(O)CC(N1CCCC1)C(=O)c2ccc3OCOc3c2	
pE1.1	Alkyl hydroxylation + α-Hydroxylation + pyrrolidine ring opening	$C_{17}H_{23}NO_5$	L	CC(C)(O)CC(NCCCC=O)C(=O)c1ccc2OCOc2c1	=pE4.1
pE1.2	Oxidative N-Dealkylation + Hydroxylation	$C_{13}H_{14}NO_5$	L	CC(C)(O)CC(=O)C(=O)c1ccc2OCOc2c1	=pE3.1, pE3.2
pE2	*N*-Dealkylation	C_4H_9N	L	C1CCCN1	
pE2.1	*N*-Dealkilation + Oxidative *N*-Dealkylation	C_4H_9NO	L	NCCCC=O	
pE3	Oxidative *N*-Dealkylation	$C_{13}H_{14}NO_4$	L	CC(C)CC(=O)C(=O)c1ccc2OCOc2c1	
pE3.1	Oxidative *N*-Dealkylation + Hydroxylation	$C_{13}H_{14}NO_5$	NL	CC(CO)CC(=O)C(=O)c1ccc2OCOc2c1	=pE1.2, pE3.2
pE3.2	Oxidative *N*-Dealkylation + Hydroxylation	$C_{13}H_{14}NO_5$	NL	CC(C)C(O)C(=O)C(=O)c1ccc2OCOc2c1	=pE1.2, pE3.1
pE4	α-Hydroxylation + pyrrolidine ring opening	$C_{17}H_{23}NO_4$	L	CC(C)CC(NCCCC=O)C(=O)c1ccc2OCOc2c1	=pG1, pG2, pG5, pG8, pG10
pE4.1	α-Hydroxylation + pyrrolidine ring opening + Hydroxylation	$C_{17}H_{23}NO_5$	L	CC(C)CC(NCCCC(=O)O)C(=O)c1ccc2OCOc2c1	=pE1.1
pBT1	β-Ketoreduction	$C_{17}H_{25}NO_3$	NA	CC(C)CC(N1CCCC1)C(O)c1ccc2OCOc2c1	=pG3, pG7
pBT1.1	β-Ketoreduction + *O*-Glucuronidation	$C_{23}H_{33}NO_9$	NA	O=C(O)C1OC(OC(C(CC(C)C)N2CCCC2)c2ccc3OCOc3c2)C(O)C(O)C1O	
pBT1.2	β-Ketoreduction + *O*-Sulfation	$C_{17}H_{25}NO_6S$	NA	OS(=O)(=O)OC(C(CC(C)C)N1CCCC1)c1ccc2OCOc2c1	

L, likely; NA, not applicable; NL, not likely; pBT, predicted by BioTransformer; pE, predicted by EAWAG-BBP; pG, predicted by GLORYx

Table S3. Proposed metabolic reaction, elemental composition, accurate mass of the molecular ion, retention time, peak area of MDPiHP and metabolites in pHLM.

ID	Proposed metabolic reaction	Elemental composition	$[M+H]^+$	RT (min)	peak area		
					pHLM 30 min	pHLM 1h	pHLM 2h
MDPiHP		$C_{17}H_{24}NO_3$	290.1751	5.6	5.5×10^7	5.0×10^7	5.0×10^7
M1	β-Keto reduction (Dihydro-MDPiHP)	$C_{17}H_{26}NO_3$	292.1907	5.5	3.6×10^5	5.1×10^5	2.5×10^5
M1.2	β-Keto reduction (Dihydro-MDPiHP)	$C_{17}H_{26}NO_3$	292.1907	5.2	6.8×10^6	7.8×10^6	3.2×10^6
M1 *O*-Gluc	β-Keto reduction (Dihydro-MDPiHP) + *O*-Glucuronidation	$C_{23}H_{34}NO_9$	468.2228	4.4	ND	ND	ND
M2	β-Hydroxylation on pyrrolidine ring	$C_{17}H_{24}NO_4$	306.1700	5.5	5.8×10^5	6.8×10^5	3.3×10^5
M2.1	Aliphatic hydroxylation	$C_{17}H_{24}NO_4$	306.1700	4.4	ND	ND	ND
M2.2	β-Hydroxylation on pyrrolidine ring	$C_{17}H_{24}NO_4$	306.1700	5.4	ND	ND	ND
M2.3	β-Hydroxylation on pyrrolidine ring	$C_{17}H_{24}NO_4$	306.1700	5.7	9.7×10^5	3.9×10^5	3.8×10^5
M2.4	β-Hydroxylation on pyrrolidine ring	$C_{17}H_{24}NO_4$	306.1700	6.0	1.3×10^5	3.7×10^5	3.0×10^5
M2.5	β-Hydroxylation on pyrrolidine ring	$C_{17}H_{24}NO_4$	306.1700	6.3	3.7×10^5	1.4×10^5	1.3×10^5
M2.6	β-Hydroxylation on pyrrolidine ring	$C_{17}H_{24}NO_4$	306.1700	6.9	2.5×10^6	2.8×10^6	3.5×10^6
M2 *O*-Gluc	Aliphatic hydroxylation + *O*-Glucuronidation	$C_{23}H_{32}NO_{10}$	482.2021	4.5	ND	ND	ND
M3	α-Hydroxylation + Pyrrolidine ring opening + Carboxylation	$C_{17}H_{24}NO_5$	322.1649	5.8	3.0×10^5	2.7×10^5	2.3×10^5
M4	α-Hydroxylation + Pyrrolidine ring opening + Carboxylation + Demethylenation + *O*-Methylation	$C_{17}H_{26}NO_5$	324.1805	4.9	ND	ND	ND
M5	α-Hydroxylation + Pyrrolidine ring opening + Carboxylation + β-Keto reduction	$C_{17}H_{26}NO_5$	324.1805	5.6	ND	ND	ND
M6	α-Hydroxylation + Pyrrolidine ring opening + Carboxylation + Aliphatic hydroxylation	$C_{17}H_{24}NO_6$	338.1598	5.1	ND	ND	ND
M7	β-Keto reduction (Dihydro-MDPiHP) + Aliphatic hydroxylation	$C_{17}H_{26}NO_4$	308.1856	4.5	ND	ND	ND
M7.1	β-Keto reduction (Dihydro-MDPiHP) + Aliphatic hydroxylation	$C_{17}H_{26}NO_4$	308.1856	5.0	ND	ND	ND
M7.2	β-Keto reduction (Dihydro-MDPiHP) + Aliphatic hydroxylation	$C_{17}H_{26}NO_4$	308.1856	5.3	1.1×10^5	3.8×10^5	2.8×10^5
M8	*O*-Demethylenation	$C_{16}H_{24}NO_3$	278.1751	4.6	4.5×10^6	4.8×10^6	4.3×10^6

M8 *O*-Gluc	*O*-Demethylenation + *O*-Glucuronidation	$C_{22}H_{32}NO_9$	454.2072	4.4	ND	ND	ND
M9	β-Keto reduction (Dihydro-MDPiHP) + *O*-Demethylenation	$C_{16}H_{26}NO_3$	280.1907	4.2	ND	ND	ND
M10	*O*-Demethylenation + *O*-Methylation	$C_{17}H_{25}NO_3$	292.1907	4.8	ND	ND	ND
M10 *O*-Gluc	*O*-demethylenation + *O*-Methylation + *O*-Glucuronidation	$C_{23}H_{34}NO_9$	468.2228	4.1	ND	ND	ND
M10.1 *O*-Gluc	*O*-demethylenation + *O*-Methylation + *O*-Glucuronidation	$C_{23}H_{34}NO_9$	468.2228	4.2	ND	ND	ND
M11	β-Oxidation on pyrrolidine ring	$C_{17}H_{22}NO_4$	304.1543	9.4	7.8×10^5	2.0×10^6	2.1×10^6

ND, not detected; pHLM, pooled human microsomes; RT, retention time.

Table S4. Proposed metabolic reaction, elemental composition, accurate mass of the molecular ion, retention time, peak area of MDPiHP and metabolites in human samples.

ID	Proposed metabolic reaction	Elemental composition	$[M+H]^+$	RT (min)	peak area (conc. ng/mL)		peak area (conc. ng/mL)		peak area (conc. ng/mL)		peak area (conc. ng/mL)	peak area (conc. ng/mL)	peak area (conc. ng/mL)	peak area (conc. ng/mL)	peak area (conc. ng/mL)
					U #1	FB #1	U #2	S #2	U #3	FB #3	S #4	FB #5	S #6	S #7	P #8
MDPiHP		$C_{17}H_{24}NO_3$	290.1751	5.6	5.2 x 10^{7} (D)	1.9 x 10^{7} (34)	6.9 x 10^{6} (12)	5.1 x 10^{5} (1.5)	4.5 x 10^{7} (D)	5.9 x 10^{7} (26)	1.1 x 10^{7} (3.0)	6.1 x 10^{6} (5.2)	3.0 x 10^{7} (6.3)	4.4 x 10^{7} (13)	2.6 x 10^{7} (11)
M1	β-Keto reduction (Dihydro-MDPiHP)	$C_{17}H_{26}NO_3$	292.1907	5.5	ND	6.8 x 10^{5}	ND	ND	ND	ND	ND	ND	ND	ND	ND
M1.2	β-Keto reduction (Dihydro-MDPiHP)	$C_{17}H_{26}NO_3$	292.1907	5.2	6.4 x 10^{7}	2.7 x 10^{7}	3.5 x 10^{7}	1.8 x 10^{6}	5.4 x10^{7}	3.0 x 10^{7}	2.0 x 10^{6}	2.9 x 10^{7}	1.4 x 10^{7}	3.5 x 10^{7}	2.4 x 10^{7}
M1 *O*-Gluc	β-Keto reduction (Dihydro-MDPiHP) + *O*-Glucuronidation	$C_{23}H_{34}NO_9$	468.2228	4.4	7.0 x 10^{5}	ND	ND	ND	ND	ND	ND	ND	ND	ND	ND
M2	β-Hydroxylation on pyrrolidine ring	$C_{17}H_{24}NO_4$	306.1700	5.5	ND	ND	ND	ND	ND	ND	ND	ND	3.4 x 10^{5}	ND	ND
M2.1	Aliphatic hydroxylation	$C_{17}H_{24}NO_4$	306.1700	4.4	1.4 x 10^{6}	ND	ND	ND	1.3 x 10^{6}	ND	ND	ND	ND	5.2 x 10^{5}	4.6 x 10^{5}
M2.2	β-Hydroxylation on pyrrolidine ring	$C_{17}H_{24}NO_4$	306.1700	5.4	2.6 x 10^{6}	1.6 x 10^{5}	ND	ND	ND	1.4 x 10^{6}	ND	ND	ND	8.7 x 10^{5}	3.0 x 10^{5}
M2.3	β-Hydroxylation on pyrrolidine ring	$C_{17}H_{24}NO_4$	306.1700	5.7	ND	ND	ND	ND	ND	ND	ND	ND	ND	ND	ND
M2.4	β-Hydroxylation on pyrrolidine ring	$C_{17}H_{24}NO_4$	306.1700	6.0	5.6 x 10^{5}	ND	ND	ND	ND	ND	ND	ND	ND	ND	ND
M2.5	β-Hydroxylation on pyrrolidine ring	$C_{17}H_{24}NO_4$	306.1700	6.3	ND	ND	ND	ND	ND	ND	ND	ND	ND	ND	ND
M2.6	β-Hydroxylation on pyrrolidine ring	$C_{17}H_{24}NO_4$	306.1700	6.9	ND	ND	ND	ND	ND	ND	ND	ND	ND	ND	ND
M2 *O*-Gluc	Aliphatic hydroxylation + *O*-Glucuronidation	$C_{23}H_{32}NO_{10}$	482.2021	4.5	6.5 x 10^{6}	ND	ND	ND	ND	ND	ND	ND	ND	ND	ND
M3	α-Hydroxylation + Pyrrolidine ring opening + Carboxylation	$C_{17}H_{24}NO_5$	322.1649	5.8	4.1 x 10^{7}	1.1 x 10^{6}	1.5 x 10^{6}	ND	4.2 x 10^{7}	1.3 x10^{6}	1.1 x 10^{6}	3.4 x 10^{5}	1.7 x 10^{6}	5.1 x 10^{6}	2.9 x 10^{6}

M4	α-Hydroxylation + Pyrrolidine ring opening + Carboxylation + Demethylenation + *O*-Methylation	$C_{17}H_{26}NO_5$	324.1805	4.9	5.9 x 10^5	ND	ND	ND	8.7 x 10^5	ND	ND	ND	ND	ND	ND
M5	α-Hydroxylation + Pyrrolidine ring opening + Carboxylation + β-Keto reduction	$C_{17}H_{26}NO_5$	324.1805	5.6	2.7 x 10^5	ND	ND	ND	ND	ND	ND	ND	ND	ND	ND
M6	α-Hydroxylation + Pyrrolidine ring opening + Carboxylation + Aliphatic hydroxylation	$C_{17}H_{24}NO_6$	338.1598	5.1	1.5 x 10^6	ND	ND	ND	ND	ND	ND	ND	ND	ND	ND
M7	β-Keto reduction (Dihydro-MDPiHP) + Aliphatic hydroxylation	$C_{17}H_{26}NO_4$	308.1856	4.5	7.4 x 10^6	5.7 x 10^4	1.8 x 10^6	ND	4.5 x 10^6	ND	7.3 x 10^4	ND	ND	ND	ND
M7.1	β-Keto reduction (Dihydro-MDPiHP) + Aliphatic hydroxylation	$C_{17}H_{26}NO_4$	308.1856	5.0	ND	ND	6.8 x 10^5	ND	2.0 x 10^5	ND	ND	ND	ND	ND	ND
M7.2	β-Keto reduction (Dihydro-MDPiHP) + Aliphatic hydroxylation	$C_{17}H_{26}NO_4$	308.1856	5.3	ND	ND	1.7 x 10^5	ND	ND	ND	ND	ND	ND	4.0 x 10^4	ND
M8	*O*-Demethylenation	$C_{16}H_{24}NO_3$	278.1751	4.6	5.2 x 10^5	ND	ND	ND	ND	ND	ND	ND	1.1 x 10^6	5.1 x 10^5	ND
M8 *O*-Gluc	*O*-Demethylenation + *O*-Glucuronidation	$C_{22}H_{32}NO_9$	454.2072	4.4	ND	ND	ND	ND	5.0 x 10^5	ND	ND	ND	ND	ND	ND
M9	β-Keto reduction (Dihydro-MDPiHP) + *O*-Demethylenation	$C_{16}H_{26}NO_3$	280.1907	4.2	1.5 x 106	ND	ND	ND	ND	ND	ND	ND	ND	ND	ND
M10	*O*-Demethylenation + *O*-Methylation	$C_{17}H_{25}NO_3$	292.1907	4.8	3.1 x 10^6	3.6 x 10^5	ND	ND	1.2 x 10^6	8.9 x 10^5	5.1 x 10^4	1.0 x 10^6	3.0 x 10^5	1.4 x 10^6	4.6 x 10^5
M10 *O*-Gluc	*O*-demethylenation + *O*-Methylation + *O*-Glucuronidation	$C_{23}H_{34}NO_9$	468.2228	4.1	6.1 x 10^5	ND	ND	ND	3.8 x 10^5	ND	ND	ND	ND	ND	ND
M10.1 *O*-Gluc	*O*-demethylenation + *O*-Methylation + *O*-Glucuronidation	$C_{23}H_{34}NO_9$	468.2228	4.2	6.7 x 10^5	ND	ND	ND	ND	ND	ND	ND	ND	ND	ND

M11	β-Oxidation on pyrrolidine ring	$C_{17}H_{22}NO_4$	304.1543	9.4	8.6 x 10^5	ND	ND	ND	ND	ND	ND	ND	ND	ND	ND

D, Detected; FB, femoral blood; ND, not detected; P, plasma; RT, retention time; S, serum; U, urine

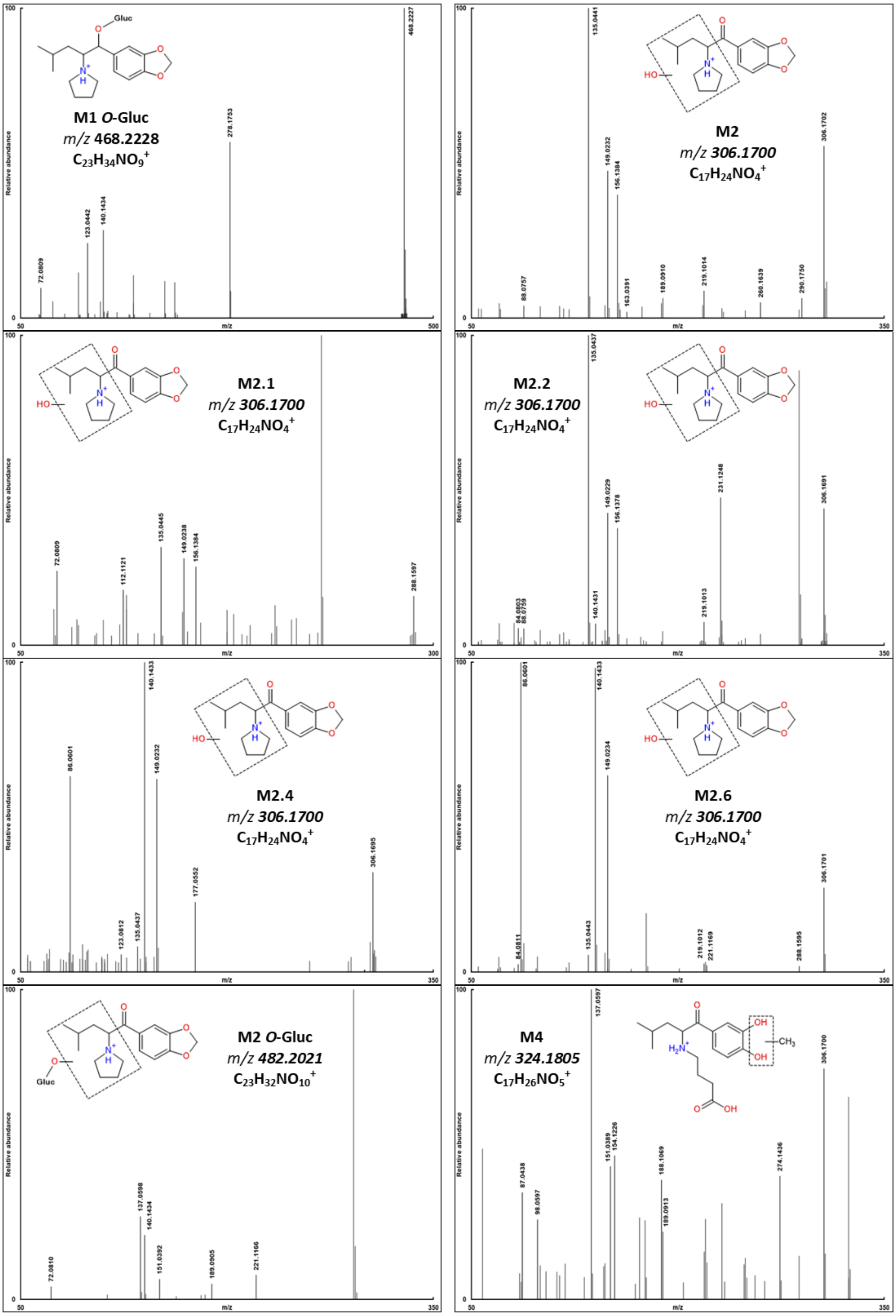

M1 O-Gluc
m/z 468.2228
$C_{23}H_{34}NO_9^+$
Gluc
72.0809
123.0442
140.1434
278.1753
468.2227
M2
m/z 306.1700
$C_{17}H_{24}NO_4^+$
88.0757
135.0441
149.0232
156.1384
163.0391
189.0910
219.1014
260.1639
290.1750
306.1702
M2.1
m/z 306.1700
$C_{17}H_{24}NO_4^+$
72.0809
112.1121
135.0445
149.0238
156.1384
288.1597
M2.2
m/z 306.1700
$C_{17}H_{24}NO_4^+$
84.0803
88.0759
135.0437
140.1431
149.0229
156.1378
219.1013
231.1248
306.1691
M2.4
m/z 306.1700
$C_{17}H_{24}NO_4^+$
86.0601
123.0812
135.0437
140.1433
149.0232
177.0552
306.1695
M2.6
m/z 306.1700
$C_{17}H_{24}NO_4^+$
84.0811
86.0601
135.0443
140.1433
149.0234
219.1012
221.1169
288.1595
306.1701
M2 O-Gluc
m/z 482.2021
$C_{23}H_{32}NO_{10}^+$
Gluc
72.0810
137.0598
140.1434
151.0392
189.0905
221.1166
M4
m/z 324.1805
$C_{17}H_{26}NO_5^+$
87.0438
98.0597
137.0597
151.0389
154.1226
188.1069
189.0913
274.1436
306.1700
Relative abundance
m/z

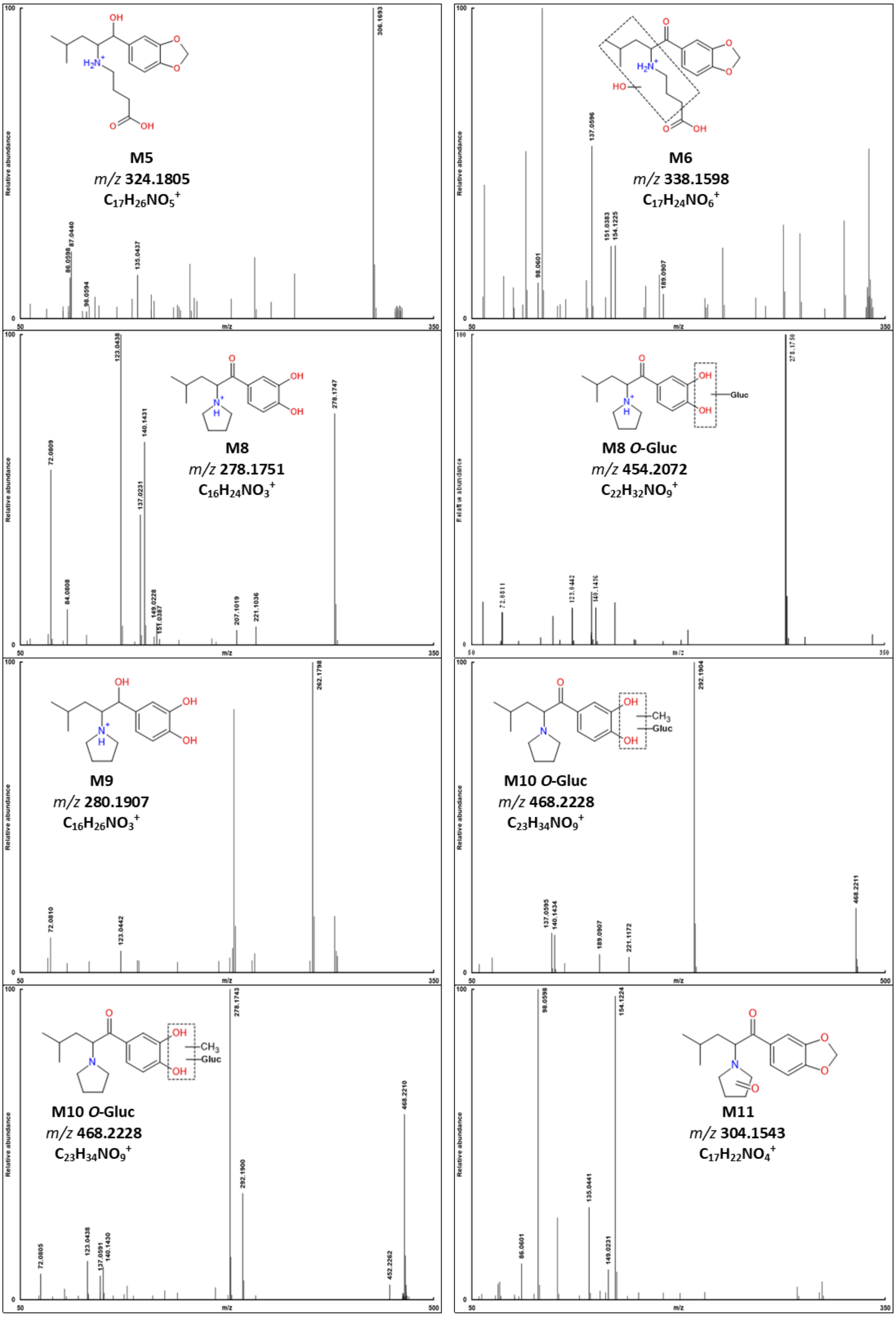

Fig.S2. Fragmentation spectra of minor *in vivo* metabolites.

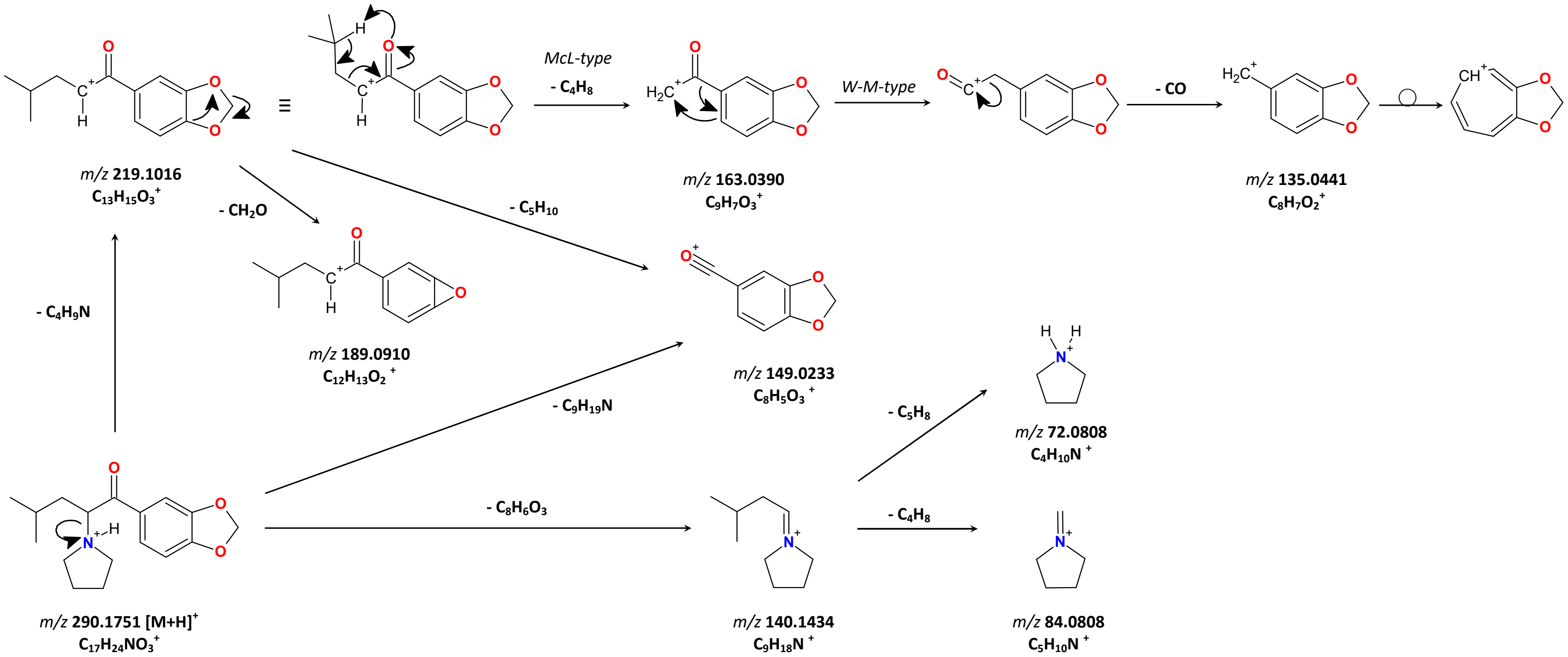

Fig. S3 Proposed fragmentation pathway for protonated **MDPiHP** based on high-resolution mass spectra acquired in positive ESI mode. Interpretation of the observed fragment ions allow the postulation of metabolite structures. Each fragment is annotated with its calculated exact mass and molecular formula, providing structural evidence to support the proposed pathway.

Abbreviations: McL-type, McLafferty-type rearrangement; W-M-type, Wagner-Meerwein-type rearrangement.

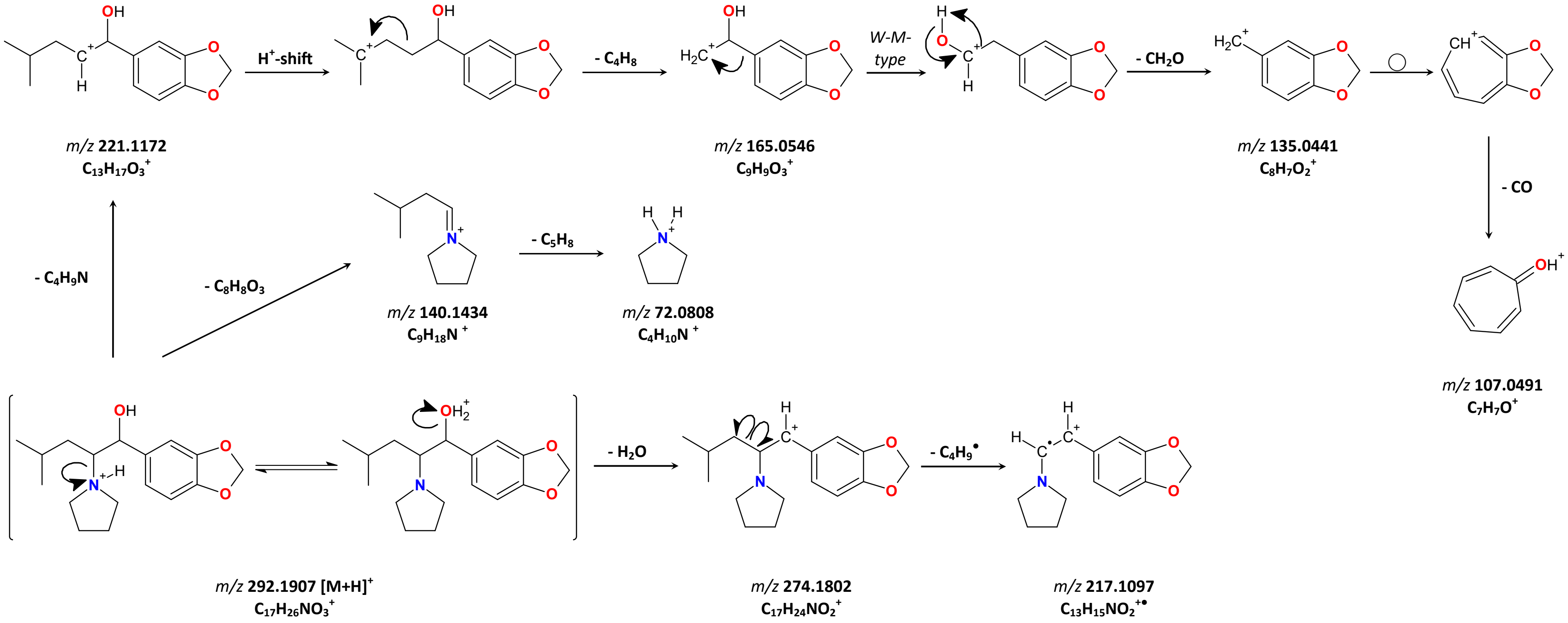

Fig. S4 Proposed fragmentation pathway of protonated metabolite **M1** and its potential diastereomer **M1.2** based on high-resolution mass spectra acquired in positive ESI mode. Interpretation of the observed fragment ions allowed the postulation of its chemical structure. Each fragment is annotated with its calculated exact mass and molecular formula, providing structural evidence to support the proposed pathway. As the stereochemistry of **M1** is unknown, this pathway should be considered for all possible diastereomers.

Abbreviation: W-M-type, Wagner-Meerwein-type rearrangement

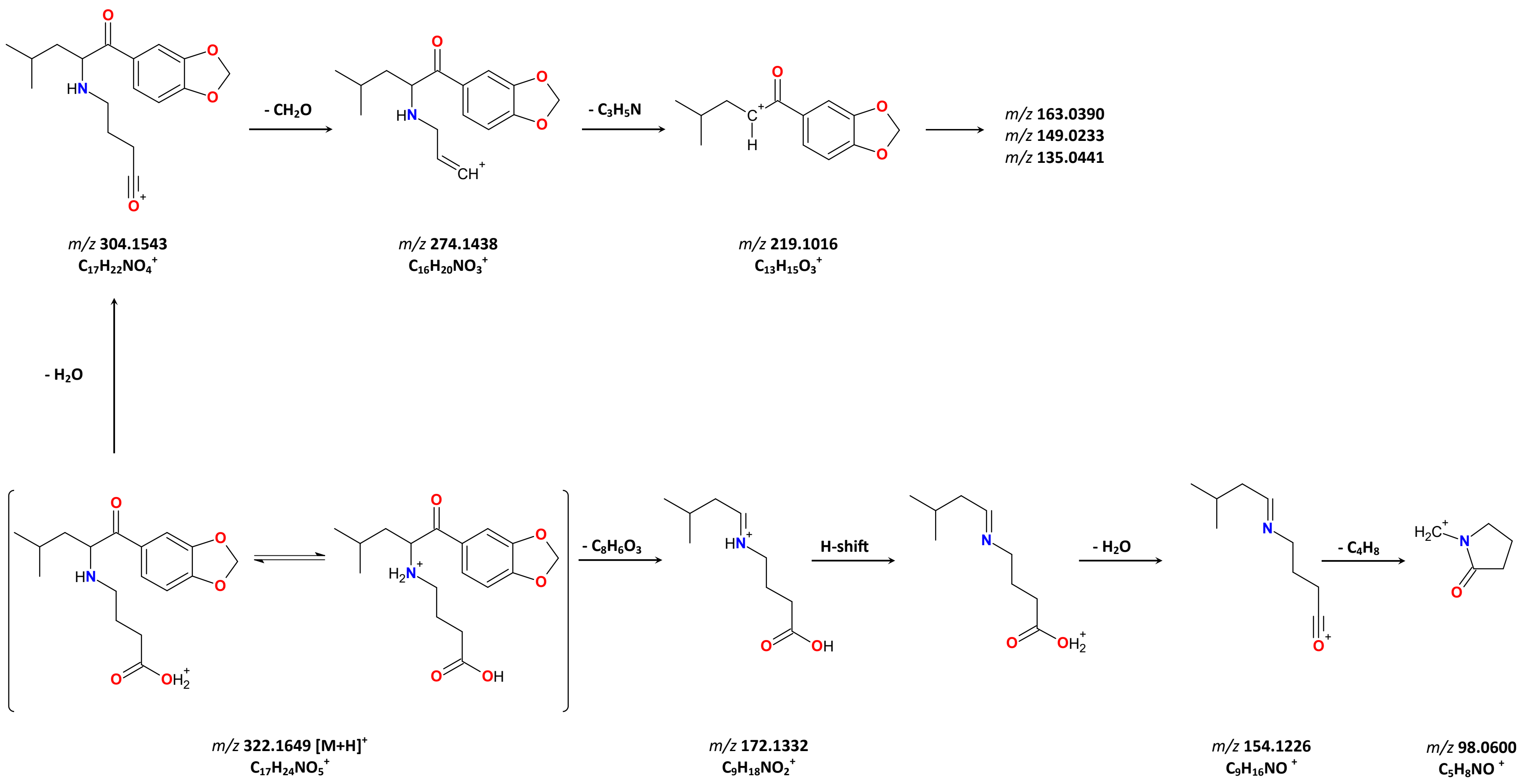

Fig. S5 Proposed fragmentation pathway of protonated metabolite **M3** based on high-resolution mass spectra acquired in positive ESI mode. Interpretation of the observed fragment ions allowed the postulation of its chemical structure. Each fragment is annotated with its calculated exact mass and molecular formula, providing structural evidence to support the proposed pathway. Subsequent fragmentations originating from *m/z* **219.1016** are detailed in Fig. **S3**.

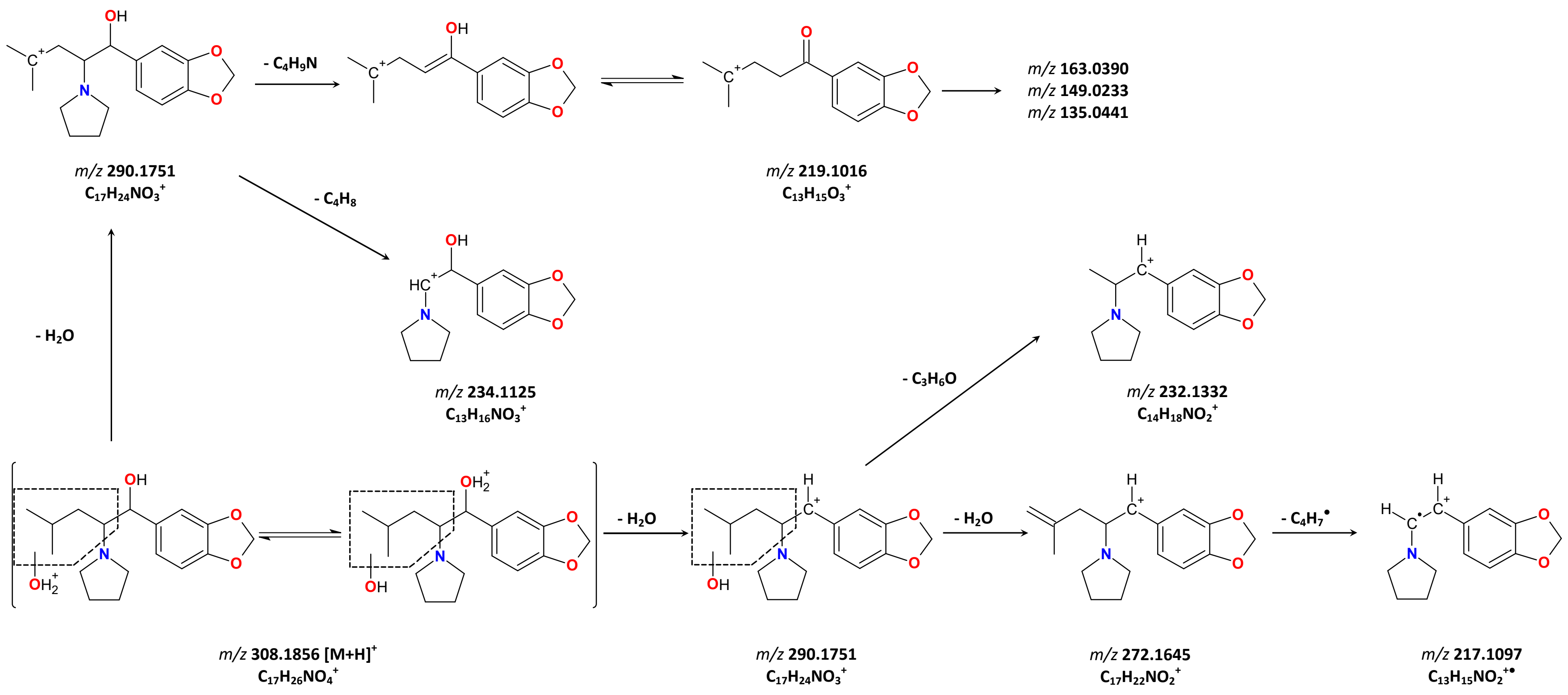

Fig. S6 Proposed fragmentation pathway of protonated metabolite **M7** and its potential isomers **M7.1** and **M7.2** based on high-resolution mass spectra acquired in positive ESI mode. Interpretation of the observed fragment ions allowed the postulation of its chemical structure. Each fragment is annotated with its calculated exact mass and molecular formula, providing structural evidence to support the proposed pathway. The double bond shown in the fragment ion at *m/z* **272.1645** represents only one of several possible isomers. Because the stereochemistry of **M7** is unknown, this fragmentation pathway should be considered for all potential isomers. Subsequent fragmentations originating from *m/z* **219.1016** are detailed in Fig. **S3**

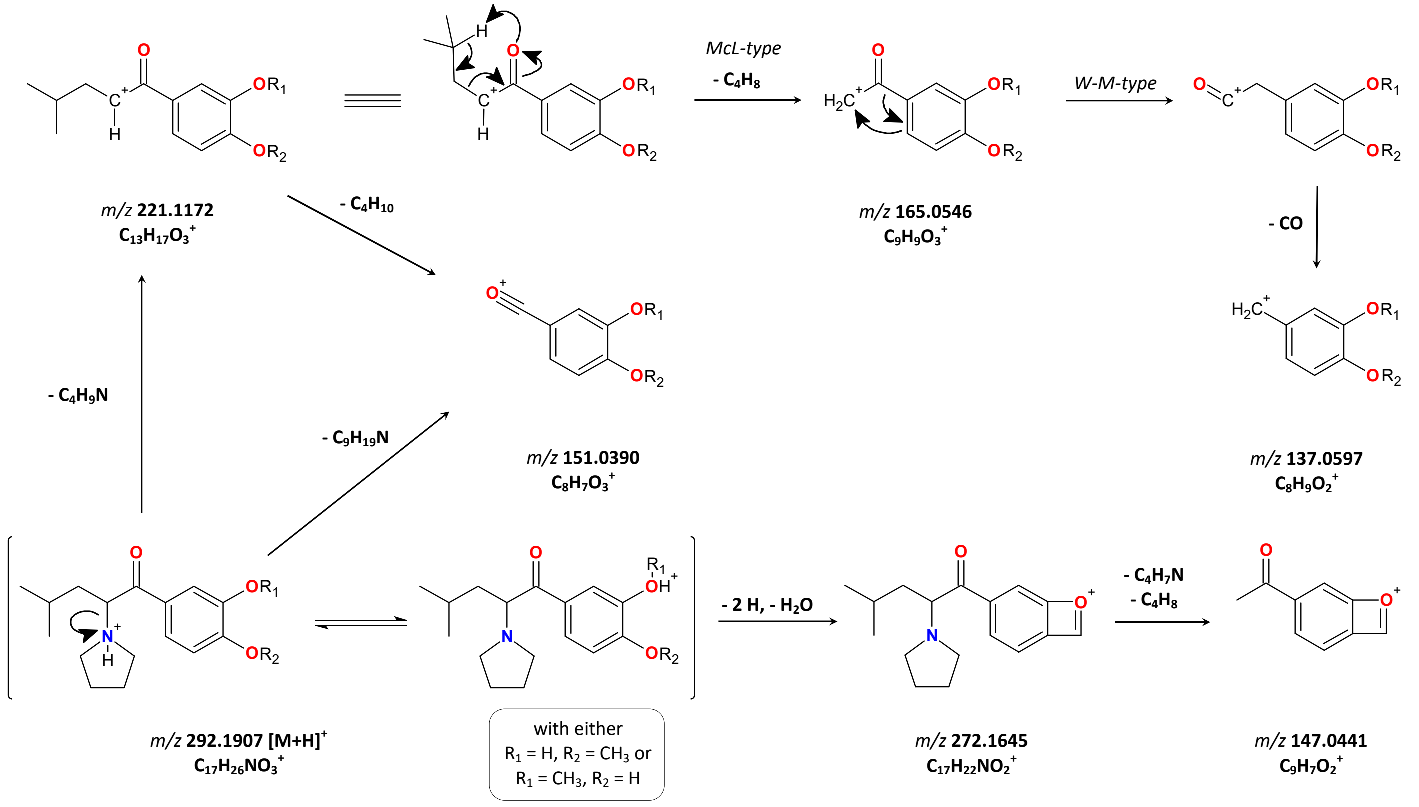

Fig. S7 Proposed fragmentation pathway of protonated metabolite **M10** based on high-resolution mass spectra acquired in positive ESI mode. The structure of **M10** incorporates aromatic substituents **OR_1** and **OR_2**, with the constraint that either **$R_1 = CH_3$** and **$R_2 = H$**, or **$R_1 = H$** and **$R_2 = CH_3$**. Interpretation of the observed fragment ions allowed the postulation of its chemical structure. Each fragment is annotated with its calculated exact mass and molecular formula, providing structural evidence to support the proposed pathway.

Abbreviations: McL-type, McLafferty-type rearrangement; W-M-type, Wagner-Meerwein-type rearrangement
